# Supplementary material for: Combined targeting of G protein‐coupled receptor and EGF receptor signaling overcomes resistance to PI3K pathway inhibitors in PTEN‐null triple negative breast cancer
Source: EMBO Mol Med. 2020 Jul 16;12(8):e11987. doi: 10.15252/emmm.202011987 (PMC7411640; doi:10.15252/emmm.202011987)
Supplement: Supplementary file 1 — Expanded View Figures PDF [file EMMM-12-e11987-s001.pdf]

Expanded View Figures

Figure EV1. A short-hairpin screening identified hits whose inhibition enhanced the effect of PI3K pathway inhibitors on PTEN-deficient triple-negative breast cancers. Related to Fig 1.

A Cell viability of cell lines treated for 4 days with 0.25 μM AZD8186, 1 μM GDC0941, or 1 μM MK2206. PTEN-deficient triple-negative breast cancer cell lines are represented by black bars, PTEN-deficient glioblastoma cell lines by black/gray dashed black, PIK3CA-mutant breast cancer cell lines by white bars, and PIK3CA WT and PTEN WT breast cancer cell lines by gray bars. Each bar represents the mean of three experiments.

B Plot of expression data extrapolated from RPKM RNAseq files of Broad Institute Cancer Cell Line Encyclopedia (CCLE, https://portals.broadinstitute.org/ccle), and specifically showing expression of PIK3CA, PIK3CB, PIK3CD, and PIK3CG across PTEN-null TNBC cell lines (namely: MDA-MB-468, HCC1395, HCC1937, HCC38, HCC70, and BT-549) and B-cell acute lymphocytic leukemia (B-cell ALL) lines (namely: BDCM, JM1, KOPN8, MHHCALL3, MHHCALL4, MUTZ5, NALM6, REH, RS411, SEM, and SUPB15). Mean ± SD. Statistical significance of two-tailed unpaired *t*-test in PIK3CD expression $^{**}P = 0.0014$ and in PIK3CG expression $^{**}P = 0.0015$.

C Cell viability of MDA-MB-468 cells infected with 10 shRNAs pools during the shRNA screen (the library was divided into 10 pools, and each dot represents one pool) and co-treated as indicated. Viability of cells was normalized to cell numbers in the vehicle-treated condition within each pool and expressed as mean ± SD.

D MDA-MB-468 was treated with serial dilutions of erlotinib or cetuximab in the presence of vehicle, AZD8186 (0.25 μM), GDC0941 (0.25 μM), or MK2206 (0.45 μM), as indicated. Cell viability was measured after 4 days (erlotinib) or 6 days (cetuximab) and normalized within each of the PI3K pathway inhibitor-treated condition to the viability in the absence of erlotinib or cetuximab. Average ± SD of triplicates and representative of two independent experiments.

E, F Viability of six PTEN-null vs five PTEN-WT TNBC cell lines treated for 6 days with pan-PI3Ki (GDC0941 1 μM) (E), or AKTi (MK2206 0.45 μM) (F), alone or in combination with EGFRi (gefitinib 3 μM). Mean of three independent experiments ± SD. Statistical significance of two-tailed unpaired Student's *t*-test in PTEN-null pan-PI3Ki vs pan-PI3Ki + EGFRi $^{**}P = 0.0041$, PTEN-null EGFRi vs pan-PI3Ki + EGFRi $^{***}P = 0.0008$, PTEN-WT pan-PI3Ki vs pan-PI3Ki + EGFRi n.s. $P = 0.166$, PTEN-WT EGFRi vs pan-PI3Ki + EGFRi n.s. $P = 0.0897$, PTEN-null AKTi vs AKTi + EGFRi $^{*}P = 0.0281$, PTEN-null EGFRi vs AKTi + EGFRi $^{***}P = 0.0059$, PTEN-WT AKTi vs AKTi + EGFRi n.s. $P = 0.1936$, PTEN-WT EGFRi vs AKTi + EGFRi n.s. $P = 0.2302$.

G Viability of three PTEN-null (MDA-MB-468, HCC70 and HCC1937), three PTEN-WT (MDA-MB-231, MDA-MB-157, and HCC1428) and two PIK3CA-mutant (SUM159 and BT20) TNBC cell lines treated for 6 days with PI3Kβi (AZD8186 90 nM) (left), or PI3Kαi (BYL719 1.2 μM) (right), alone or in combination with EGFRi (gefitinib 3 μM). Mean of 3–4 independent experiments ± SD. Statistical significance of two-tailed unpaired Student's *t*-test PI3Kbi + EGFR treatments in PTEN-null vs PTEN-WT $^{*}P = 0.0158$, PI3Kai + EGFR treatments in PTEN-null vs PTEN-WT n.s. $P = 0.1081$.

H Dot-plot showing the fold change (log_2) in number of reads between vehicle and AZD8186-treated conditions vs the *P*-value of the difference between the two treatment conditions for each shRNA. Three out of four shRNAs targeting CSNK2A2 and three out of seven shRNAs targeting CSNK2B showed a *P*-value < 0.2 calculated by two-sided paired *t*-test and are highlighted in the figure.

I, J MDA-MB-468 was infected with the indicated shRNAs targeting CSNK2B and selected by puromycin. CSNK2B mRNA was then measured by RT–qPCR (I), and cell viability was measured after 4 days of treatment with serial dilutions of AZD8186 (J) Average ± SD of triplicates and representative of three independent experiments.

K MDA-MB-468 cells were treated with serial dilutions of AZD8186 in combination with vehicle or with the indicated concentrations of CX4945. Viability was measured after 6 days and normalized within each of the CX4945-treated condition to the viability in the absence of AZD8186. Mean ± SD of triplicates and representative of three independent experiments.

L–N Viability of six PTEN-null vs five PTEN-WT TNBC cell lines treated for 6 days with PI3Kβi (AZD8186 10 nM) (L), AKTi (MK2206 0.45 μM) (M), or pan-PI3Ki (GDC0941 1 μM) (N) alone or in combination with CK2i (CX4945 1 μM). Mean of 3–5 independent experiments ± SD. Statistical significance calculated by two-tailed unpaired Student's *t*-test. PI3KBi vs PI3KBi + CK2i $^{*}P = 0.0265$, CK2i vs PI3KBi + CK2i $^{*}P = 0.0128$; AKTi vs AKTi + CK2i $^{*}P = 0.0494$, CK2i vs AKTi + CK2i $^{****}P < 0.0001$; pan-PI3Ki vs pan-PI3Ki + CK2i $^{*}P = 0.0235$, CK2i vs pan-PI3Ki + CK2i $^{****}P < 0.0001$; n.s. $P > 0.05$.

© 2020 The Authors

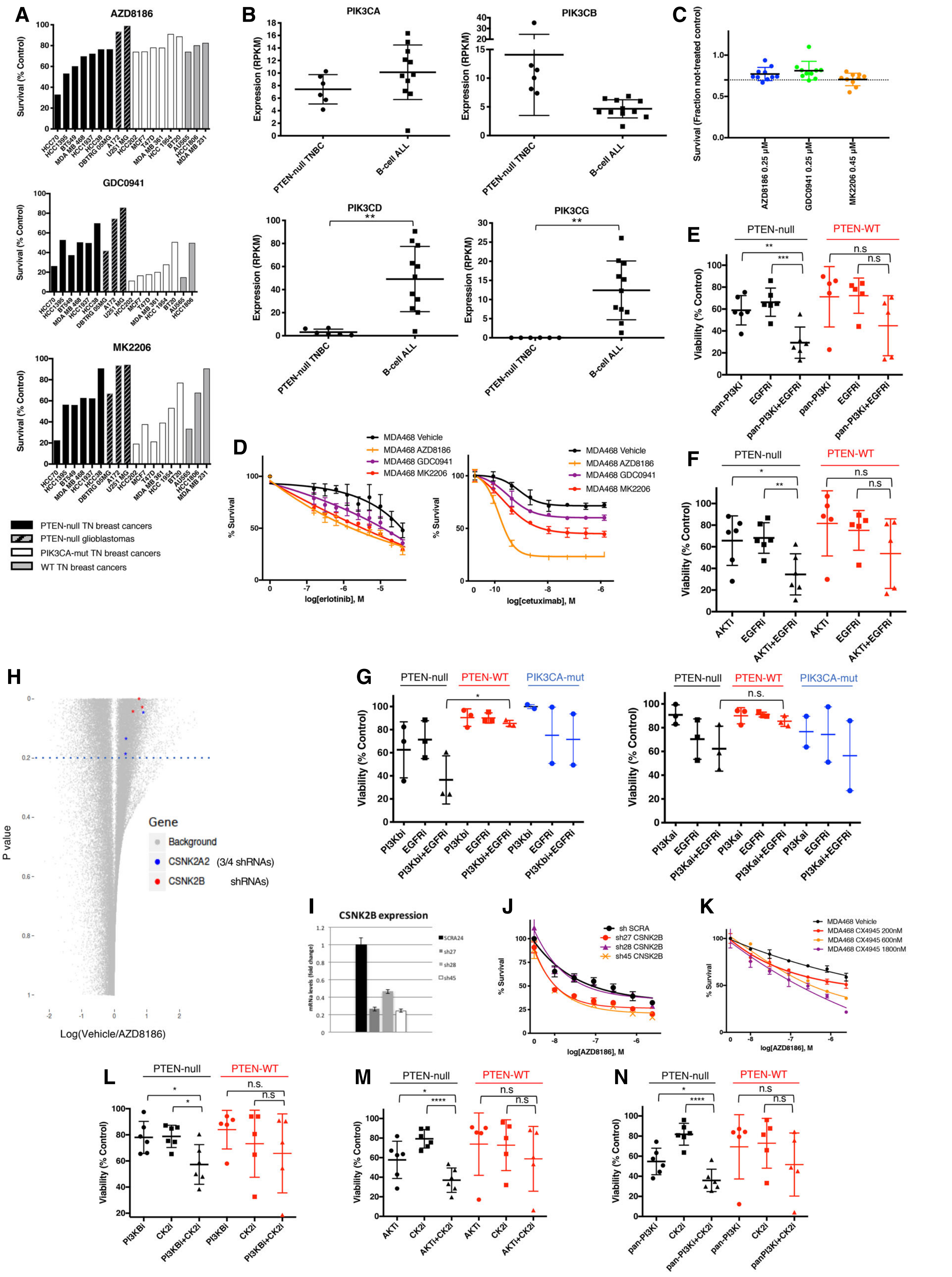

Figure EV1.

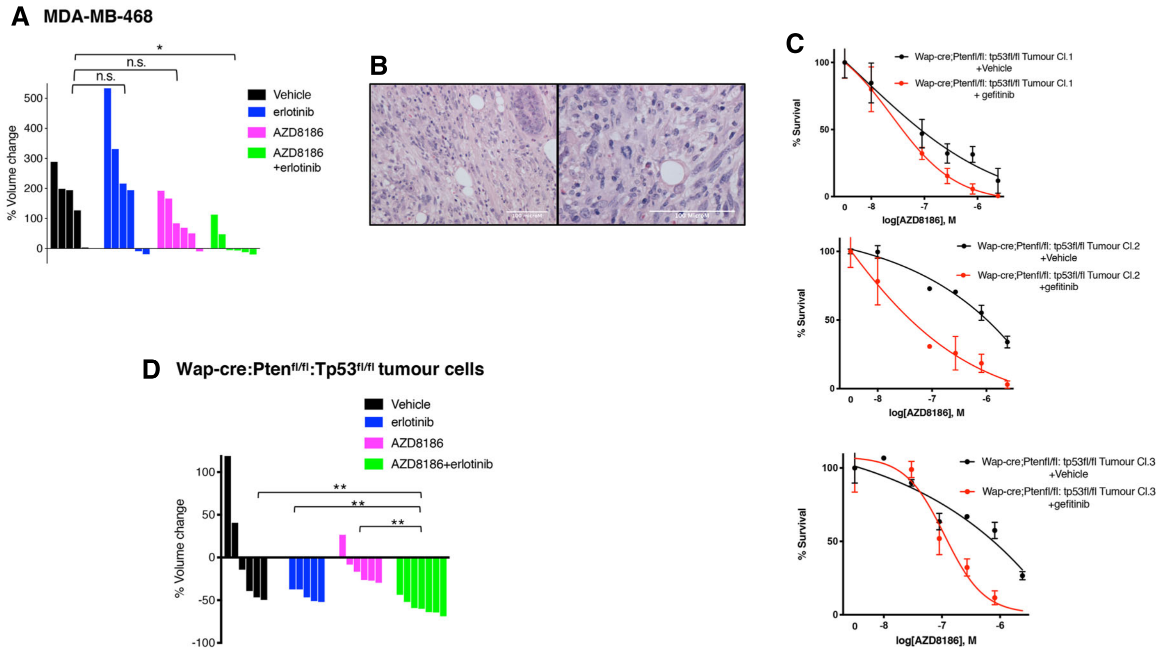

Figure EV2. Anti-tumor effects of combinatorial inhibition of PI3Kβ and EGFR on triple-negative breast cancers *in vivo*. Related to Fig 2.

A Waterfall representation of changes in the volume of individual MDA-MB-468 tumors during the indicated treatments. Statistical comparison between different treatment groups was done by Mann–Whitney test, $*P = 0.0173$, n.s. $P > 0.05$.

B H&E (Hematoxylin and Eosin) staining of sections from the mammary tumor derived from a Wap-cre:Pten$^{fl/fl}$:Tp53$^{fl/fl}$ mouse and showing spindle-shaped tumor cells. Two different magnifications of sections are shown in the panels.

C Viability of three clonal cell lines from Wap-cre:Pten$^{fl/fl}$:Tp53$^{fl/fl}$ primary mammary tumor treated with serial dilutions of AZD8186 in combination with vehicle or gefitinib 400 nM. Mean ± SD of triplicates and representative of three independent experiments.

D A clonal cell line derived from Wap-cre:Pten$^{fl/fl}$:Tp53$^{fl/fl}$ primary mammary tumor was transplanted in the mammary fat pad of syngeneic C57BL6/J mice and soon after tumor engraftment all transplanted mice, independently from the growth rate of the individual tumors, were treated with vehicle, AZD8186 (150 mg/kg, og once/day), erlotinib (50 mg/kg IP once/day) alone, or in combination (6–7 mice per group, mean ± SEM) for 19 days. Waterfall representation of changes in the volume of individual tumors during the treatments. Statistical significance of Mann–Whitney test in vehicle vs AZD8186 + erlotinib $**P = 0.0047$, erlotinib vs AZD8186 + erlotinib $**P = 0.0093$, AZD8186 vs AZD8186 + erlotinib $**P = 0.0012$.

 © 2020 The Authors

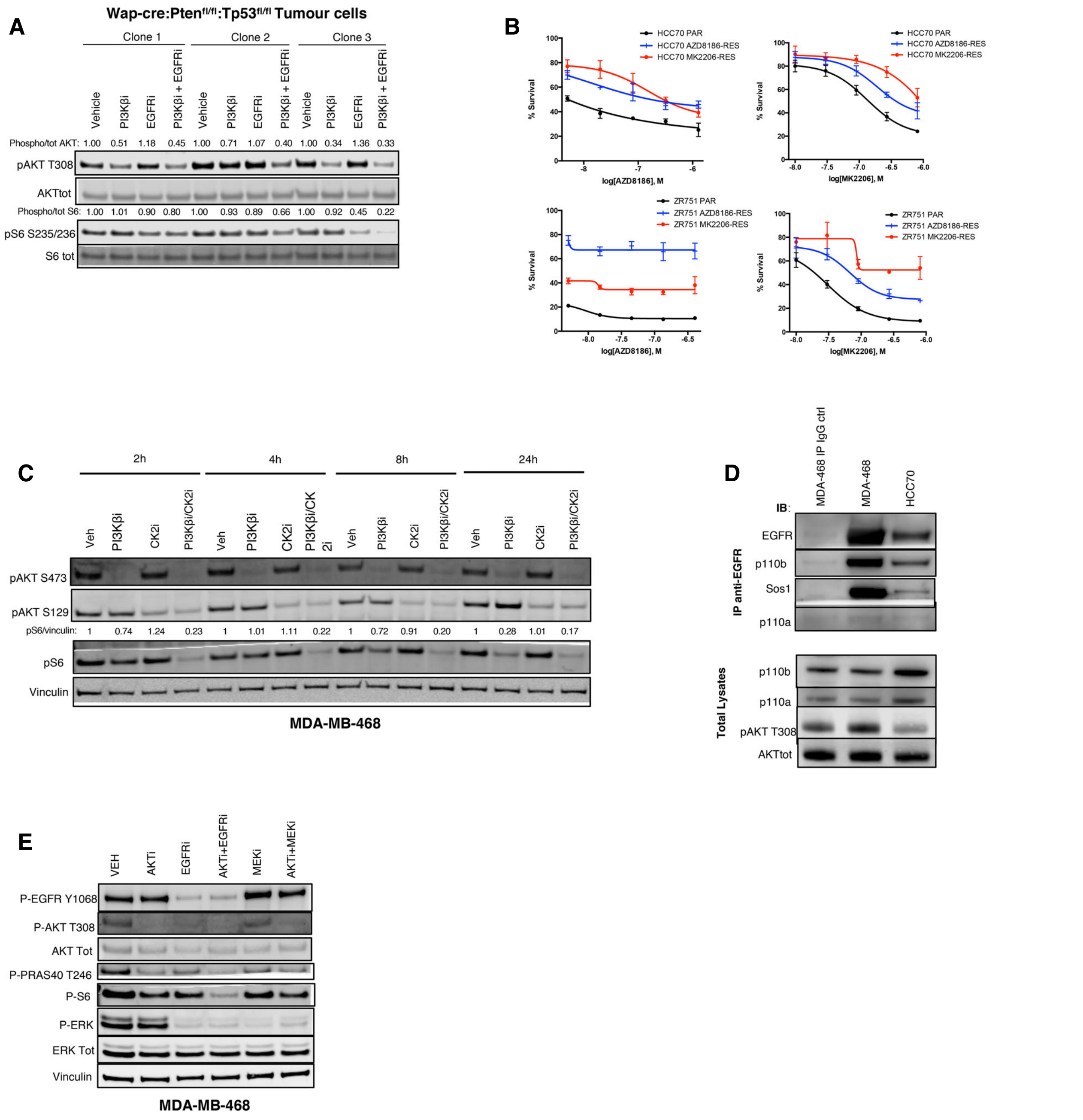

Figure EV3. Biochemical effects of EGFR and PI3K pathway combined inhibition. Related to Fig 3.

A Three cell lines derived and cloned from a mammary tumor arisen in a Wap-cre:Pten$^{fl/fl}$:Tp53$^{fl/fl}$ mouse were treated for 16 h with vehicle, PI3Kβi (AZD8186 250 nM), or EGFRi (gefitinib 3 μM), alone or in combination. The cell lysates were probed with the indicated antibodies. Quantification of the bands was performed by ImageLite software.

B Viability of HCC70 or ZR-75-1 parental cells (Par) and their respective acquired-resistant derivatives (AZD8186-Res or MK2206-Res) treated for 18–21 days with serial dilutions of AZD8186 or MK2206, as indicated, and stained by crystal violet. The staining was then dissolved in 10% acetic acid and 100 μl transferred in a 96-well plate for absorbance reading at 595 nm. Average ± SD of triplicates and representative of two independent experiments.

C Time-course analysis of MDA-MB-468 cells treated for the indicated times with vehicle, PI3Kβi (AZD8186 250 nM), or CK2i (CX4945 2 μM), alone or in combination. The cell lysates were probed with the indicated antibodies. AKT S129 is a known phosphorylation target of CK2, and the antibody raised against this phosphorylation site was used as a marker of CK2 activity. Quantification of the bands was performed by ImageLite software.

D p110β co-immunoprecipitates with EGFR. Total lysates from MDA-MB-468 or HCC70 were incubated with mouse IgG control or anti-EGFR antibody, as indicated. The immuno-precipitated complexes or the total lysates where probed with the indicated antibodies.

E MDA-MB-468 cells were treated for 24 h with vehicle, AKTi (MK2206 1 μM), EGFRi (gefitinib 3 μM), or MEKi (trametinib 50 nM), alone or in the indicated combinations. The cell lysates were probed with the indicated antibodies.

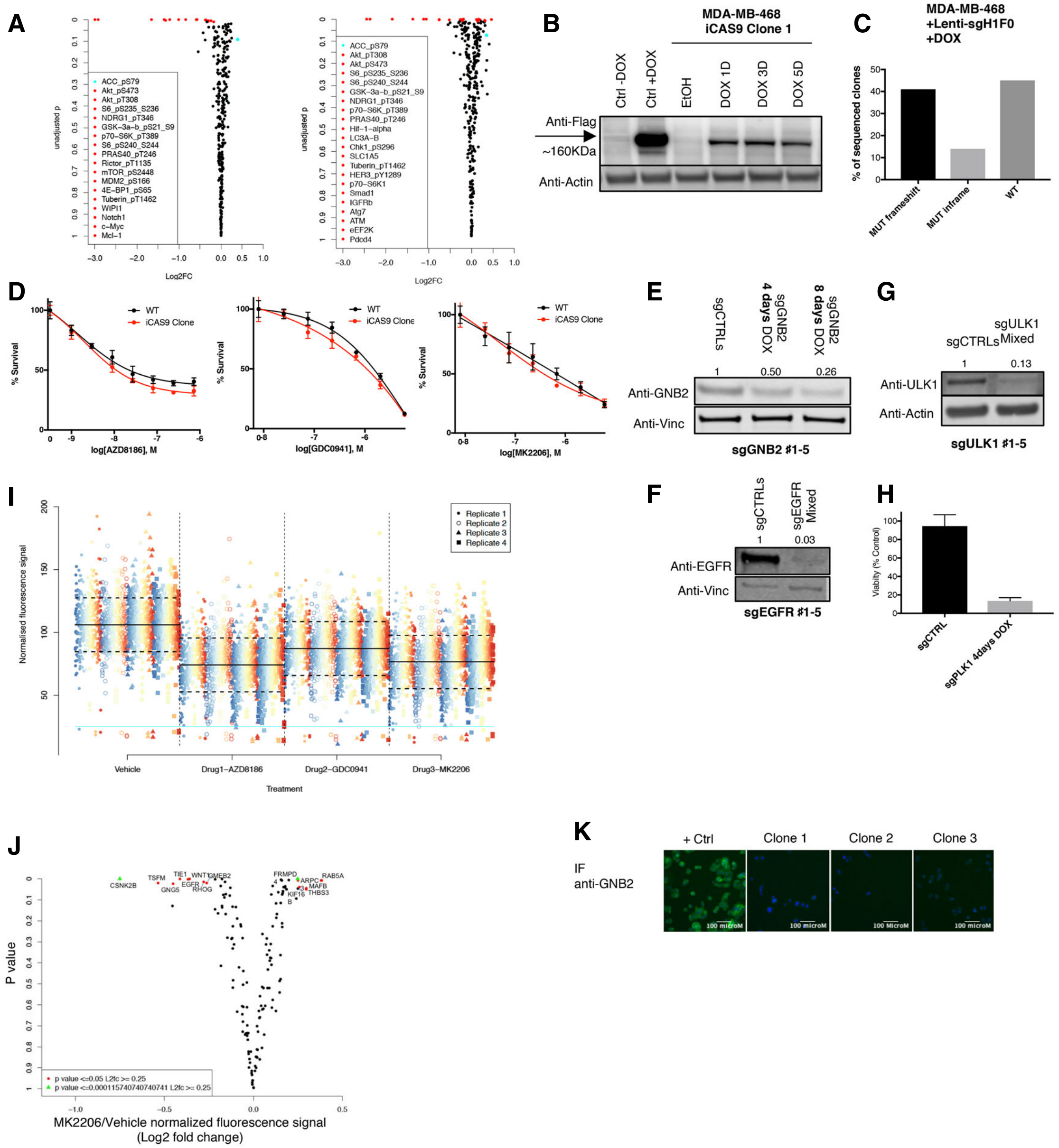

Figure EV4.

 © 2020 The Authors

Figure EV4. A CRISPR-Cas9 screening identified GNB2 as a target to potentiate the inhibition mediated by pan-PI3K inhibitor. Related to Fig 4.

A Epitopes regulated by AZD8186 treatment in MDA-MB-468 cells. Representation of the results from RPPA analysis performed with 300 antibodies: comparison of the lysates from MDA-MB-468 treated with vehicle or AZD8186 250 nM for 2 (left) or 28 h (right). The dot-plot shows the $\log_2$ fold change in signal between vehicle and treated conditions vs the *P*-value (single sample *t*-test for non-zero coefficient in the regression model) of the difference between the two conditions (biological triplicates) for each antibody.

B Inducible expression of flagged-Cas9 in MDA-MB-468. Cells transduced with the doxycycline-inducible Cas9 (iCas9) construct were selected with hygromycin and single-cell cloned. The MDA-MB-468 iCas9 clone was then treated with vehicle (ethanol) or doxycycline (DOX) for the indicated times and cell lysates probed with the indicated antibodies. Another cell line previously tested for inducible expression of Cas9 was used as a control (Ctrl), and predicted molecular weight of Cas9 is marked on the blot by an arrow.

C Frequency and types of genetic modifications induced by a single sgRNA construct in MDA-MB-468 iCas9 clone. The cells were transduced with a lentiviral construct codifying for an sgRNA that targets the gene H1F0, whose inactivation is known to do not impact cell proliferation. The target genetic locus was sequenced and the frequency of the genetic modifications reported in the bar graph.

D MDA-MB-468 parental and iCas9 clone showed similar sensitivity to PI3K pathway inhibitors. MDA-MB-468 parental cells or iCas9 clone was treated with serial dilutions of the indicated drugs for 4 days. Mean ± SD of triplicates and representative of two independent experiments.

E–G KO efficiency in MDA-MB-468 iCas9 clone. Cells were transduced with a mix of five lentiviral constructs codifying for not overlapping non-target sgRNAs (sgCTRL) or five sgRNAs designed to target GNB2 (E), EGFR (F), or ULK1 (G). Transduced cells were treated with doxycycline for the indicated times or for 8 days where not stated and cell lysates were probed with antibodies against the protein codified by the targeted gene or loading control. Quantification of the bands was performed by ImageLite software.

H Inactivation of the essential gene PLK1 in MDA-MB-468 was used here as a killing control to validate the system. MDA-MB-468 iCas9 clone was transduced with a mix of five lentiviral constructs encoding control sgRNAs or sgRNAs against PLK1, and cell viability was measured by cell titer blue after 4 days of treatment with doxycycline. Mean ± SD of two independent experiments.

I Scatter plot of raw phospho-S6 signals from CRISPR-Cas9 screening. Color corresponds to individual genes knocked-out by sgRNAs, and each dot is associated with an individual measurement from a biological and technical replicate. Technical replicates are highlighted by different shapes and positioned in separate columns within each treatment condition. Solid black line indicates the mean level across all observations per drug treatment, and dashed horizontal black lines indicate standard deviation of the same. The horizontal cyan line indicates the threshold for lower outliers. Overlap between outliers can be observed between replicates and treatment conditions

J Results of CRISPR-Cas9 screening in combination with MK2206. The dot-plots show for each gene knocked-out by sgRNAs the fold change ($\log_2$) between vehicle- and MK2206-treated condition in the fluorescence signal (anti-phosphoS6 Immunofluorescence) vs the *P*-value of the difference calculated by two-sided *t*-test. The plots represent means of biological triplicates. Genes for which it was calculated a $P < 0.0001$ and Log_2 (fold change) > 0.25 are reported and highlighted in green; genes having a $0.0001 < P < 0.05$ and a Log_2 (fold change) > 0.25 are reported and shown in red.

K MDA-MB-468 iCas9 cells transduced with a mix of four GNB2 sgRNAs were single-cell cloned after Cas9 induction and screened by immune-fluorescence (IF) with an anti-GNB2 antibody. GNB2-expressing MDA-MB-468 cells were used as a positive control (+ ctrl), and three independent clones GNB2-negative were identified (Clones 1–3). Signal from GNB2 was detected through Alexa Fluor-488 conjugate secondary antibody (green), while nucleus staining was performed by DAPI (blue signal).

© 2020 The Authors

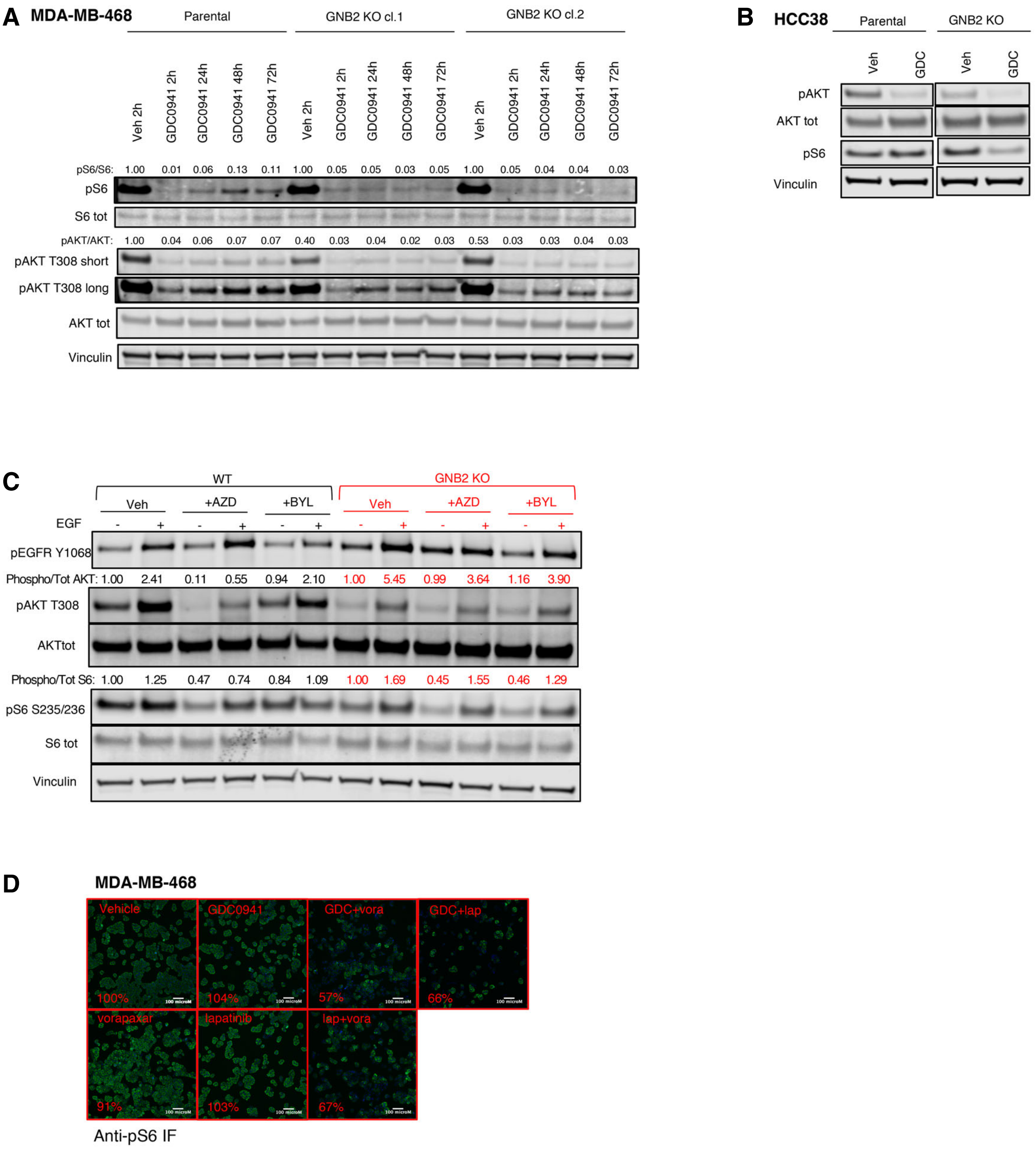

Figure EV5. GNB2 KO modifies the sensitivity to different inhibitors of EGFR-PI3K pathway. Related to Fig 5.

A MDA-MB-468 parental cells or GNB2 KO clones were treated for the indicated times with vehicle or pan-PI3K inhibitor GDC0941 450 nM. The cell lysates were probed with the indicated antibodies. Quantification of the bands was performed by ImageLite software.

B HCC38 parental or GNB2 KO cells were incubated with vehicle or GDC0941 1 µM for 24 h. Cell lysates were probed with the indicated antibodies.

C Biochemical impact of PI3K isoform-specific inhibitors on MDA-MB-468 WT and GNB2 KO. MDA-MB-468 parental or GNB2 KO cells were starved overnight, pre-treated for 1 h with vehicle, AZD8186 250 nM, or BYL719 2 µM and then stimulated with vehicle or EGF 10 ng/ml for 10 min. The cell lysates were probed with the indicated antibodies. Quantification of the bands was performed by ImageLite software.

D PAR1 signals through GNB2 to sustain the activation of PI3K pathway in the presence of PI3K or HER inhibitors. Representative immune-fluorescence stainings by phospho-S6 antibody from the GPCR compound screening performed on MDA-MB-468 and showing cells treated with the indicated compounds. pS6 green signal was from Alexa Fluor-488 conjugate secondary antibody, while nuclei stained by DAPI are shown in blue. The quantification was performed by Cellomics ArrayScan VTI high content microscopy software, and percentages of DAPI-normalized pS6 signal compared to vehicle control are reported for each treatment condition.

 © 2020 The Authors
