# Supplementary material for: Combined targeting of G protein‐coupled receptor and EGF receptor signaling overcomes resistance to PI3K pathway inhibitors in PTEN‐null triple negative breast cancer
Source: EMBO Mol Med. 2020 Jul 16;12(8):e11987. doi: 10.15252/emmm.202011987 (PMC7411640; doi:10.15252/emmm.202011987)
Supplement: Supplementary file 3 — Table EV2 [file EMMM-12-e11987-s003.pdf]

Table EV2. List of genes included in the CRISPR-CAS9 screening.

The list included top candidates from the shRNA whole-genome screening (columns on the left side) and genes codifying for epitopes regulated by AZD8186 treatment and identified by RPPA analysis (last two columns on the right). Genes not reported in bold are those at the edge of inclusion criteria that were still screened using the CRISPR-CAS9-based strategy. In red are highlighted genes that showed up as candidates from both shRNAs screening and RPPA analysis.

candidates shRNA screening						candidates RPPA analysis	
**EGFR**	**DIO2**	**ZNF202**	**RAB3B**	**PIK3CB**	SDF2L1	**GSK3A**	**CAV1**
**RHOG**	**MAP3K8**	**S100A6**	**GREB1**	**PLK1**	ADAT3	**GSK3B**	**ATM**
**CSNK2B**	**TSFM**	**PAPSS2**	**PAK6**	**UNKL**	DVL3	**RPS6KB1**	**EEF2K**
**NDNL2**	**TPSAB1**	**CLCA2**	**RNASE7**	**CDK4**	DUSP23	**Rictor**	**PRKAA1**
**RAB24**	**INSRR**	**WNT1**	**HIST1H2AB**	**MTOR**	ARPC3	**PDCD4**	**PDGFRB**
**GABARAPL2**	**NOXA1**	**YIPF7**	**FGF3**	**FANCE**	CDC25A	**DUSP4**	TRIM25
**SGK1**	**RNF145**	**ERBB4**	**MCL1**	**GRIN2A**	ZNF653	**MDM2**	IRF1
**GTSE1**	**THBS3**	**PROK1**	**ADCK1**	**ST6GALNAC1**	MSH3	**EIF4EBP1**	ATG7
**GMEB2**	**ADCY2**	**RAB11FIP1**	**NPIPA1**	**GPHN**	SSH1	**YBX1**	RELA
**TIE1**	**SLC7A1**	**WDR19**	**AKAP7**	**KIF16B**	RPTOR	**WIPI1**	ERBB3
**POU5F1**	**ATG4B**	**C2CD2L**	**CCDC64**	**BET1**		**NOTCH1**	PAK1
**CELA3A**	**C20orf95**	**HSD11B1L**	**CHEK1**	**WWC2**		**HES1**	
**OBFC2A**	**PRPSAP2**	**KLK8**	**CXXC4**	**EPHA8**		**ACACA**	
**BTBD12**	**HMCN2**	**FRMPD4**	**FLI1**	**TMEM209**		**HIF1A**	
**ITGAV**	**ULK1**	**PLA2G3**	**GATA6**	**CSPG4**		**CDK1**	
**C1orf27**	**CHST3**	**CAMKK1**	**GNB2**	**DACH2**		**CCNB1**	
**AACS**	**CTDSP1**	**MSRB3**	**HIST1H1A**	RAB5A		**MUC1**	
**RRP1B**	**GNG5**	**KLK12**	**INF2**	TMEM97		**FoxM1**	
**DNASE1L1**	**CSNK1E**	**AUP1**	**LAMP3**	ACOT7		**GJA1**	
**ARF1**	**SPIN1**	**NEUROG1**	**MFSD2A**	ITPKA		**INSR**	
