# Supplementary material for: Combined targeting of G protein‐coupled receptor and EGF receptor signaling overcomes resistance to PI3K pathway inhibitors in PTEN‐null triple negative breast cancer
Source: EMBO Mol Med. 2020 Jul 16;12(8):e11987. doi: 10.15252/emmm.202011987 (PMC7411640; doi:10.15252/emmm.202011987)

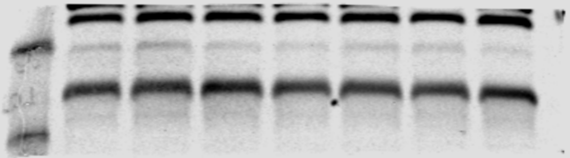

Fig 3H AKTtot total

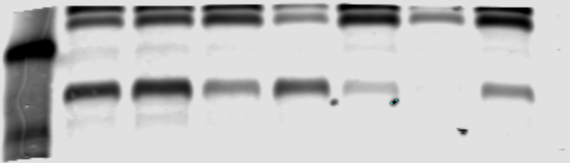

Fig 3H pAKT total

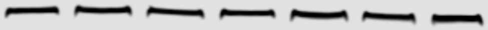

Fig 3H Vinculin total

Fig 3H IP EGFR

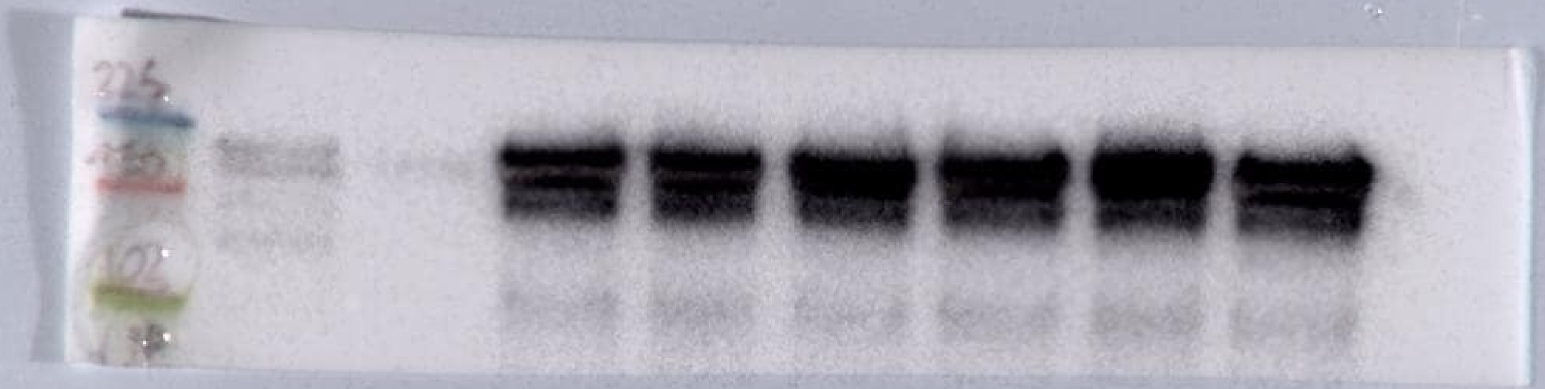

Fig 3H IP p110b

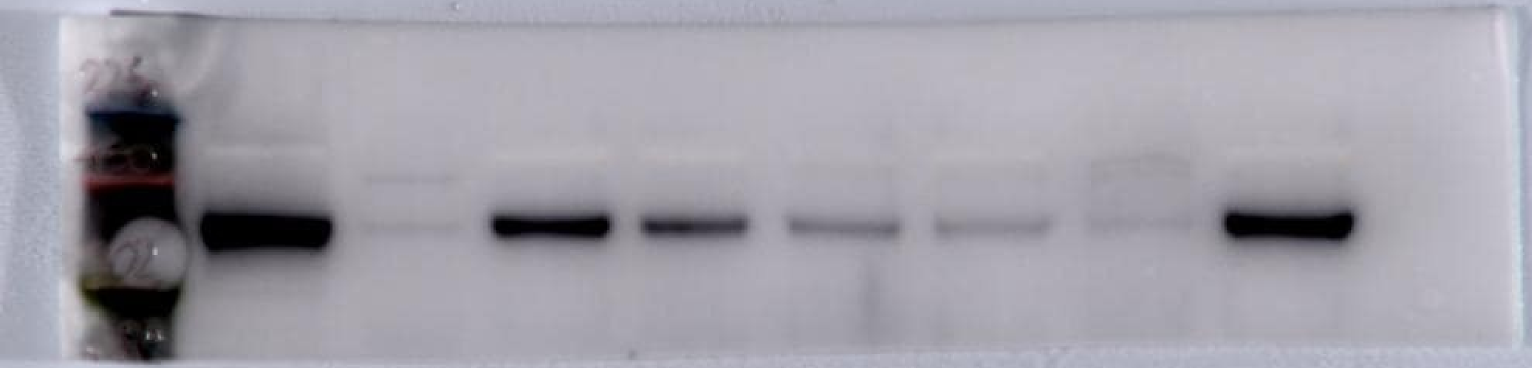

Fig 3H IP Sos1

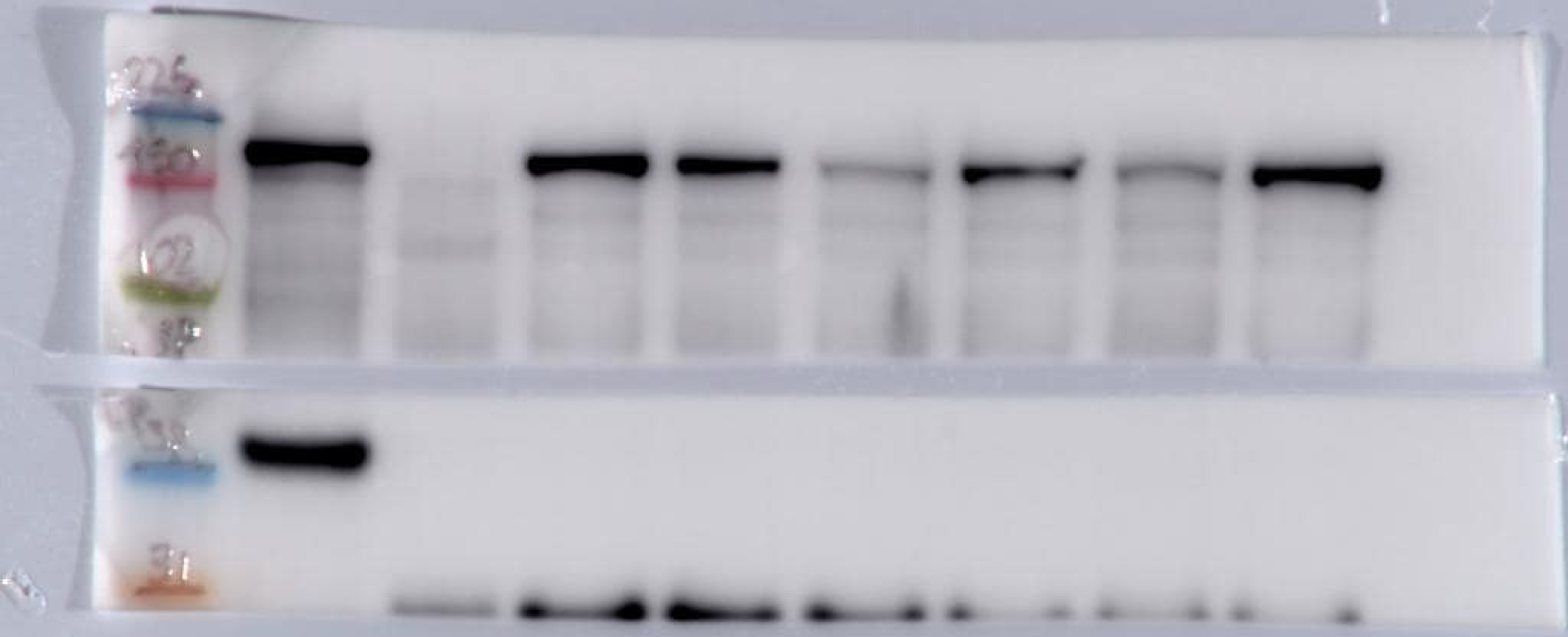

Fig 3H p110b total_bottom blot

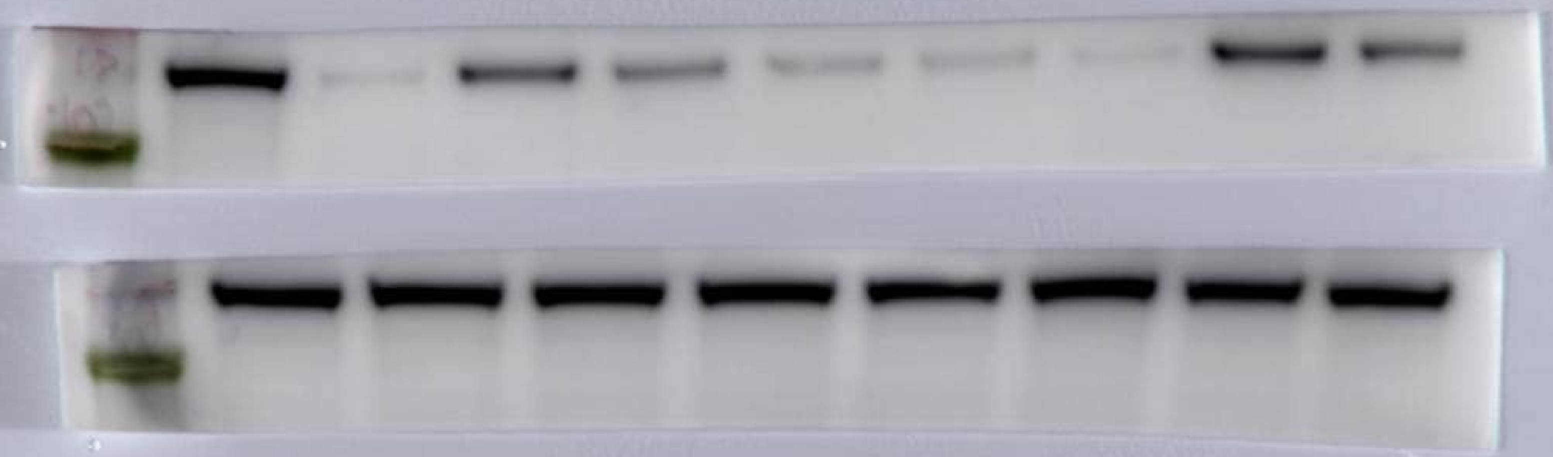

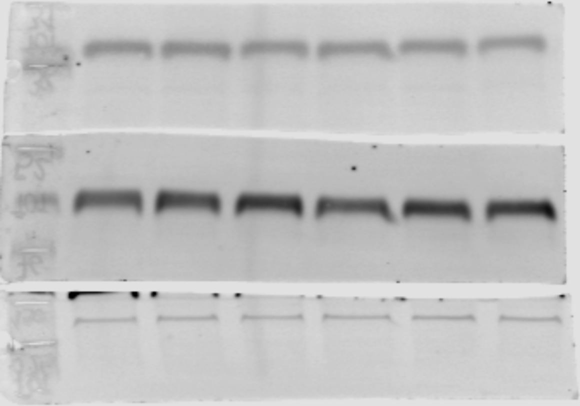

Fig 3I AKTtot_S6tot total

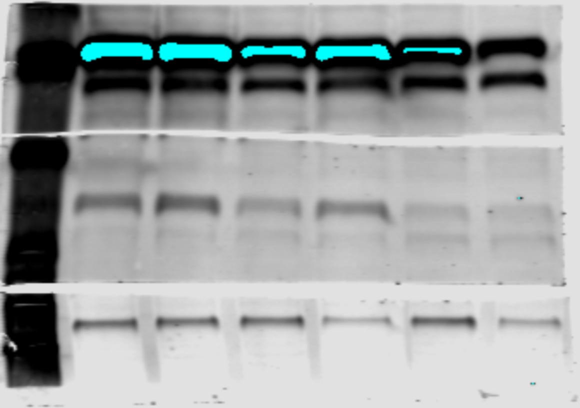

Fig 3I pAKT total

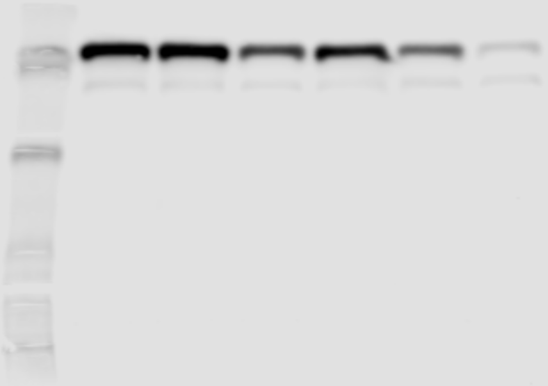

Fig 3I pS6 total

Fig 3I IP EGFR_p110b total

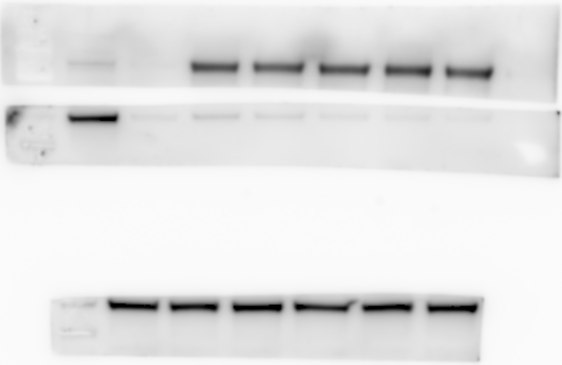

Fig 3I IP p110b

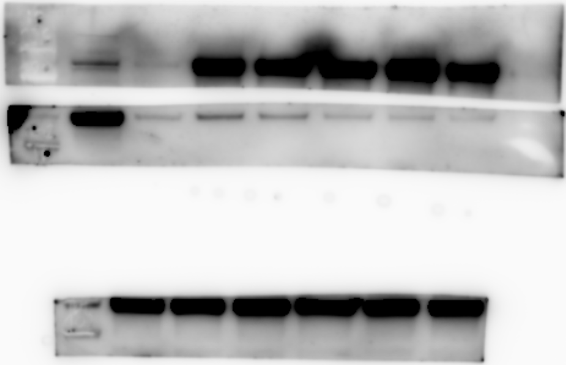

Fig 3J AKTtot_Vinculin

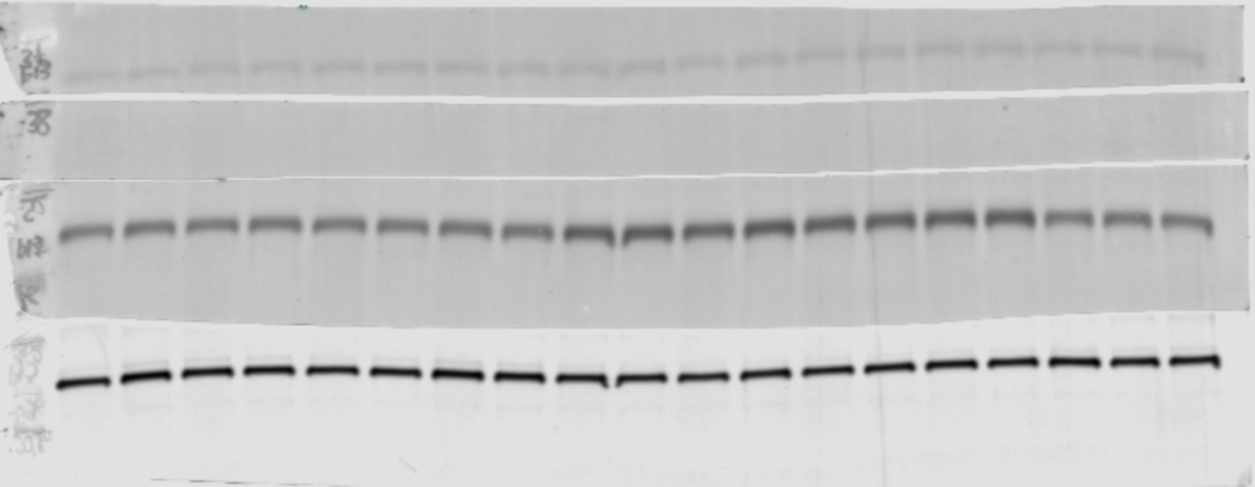

Fig 3J pAKT pPRAS

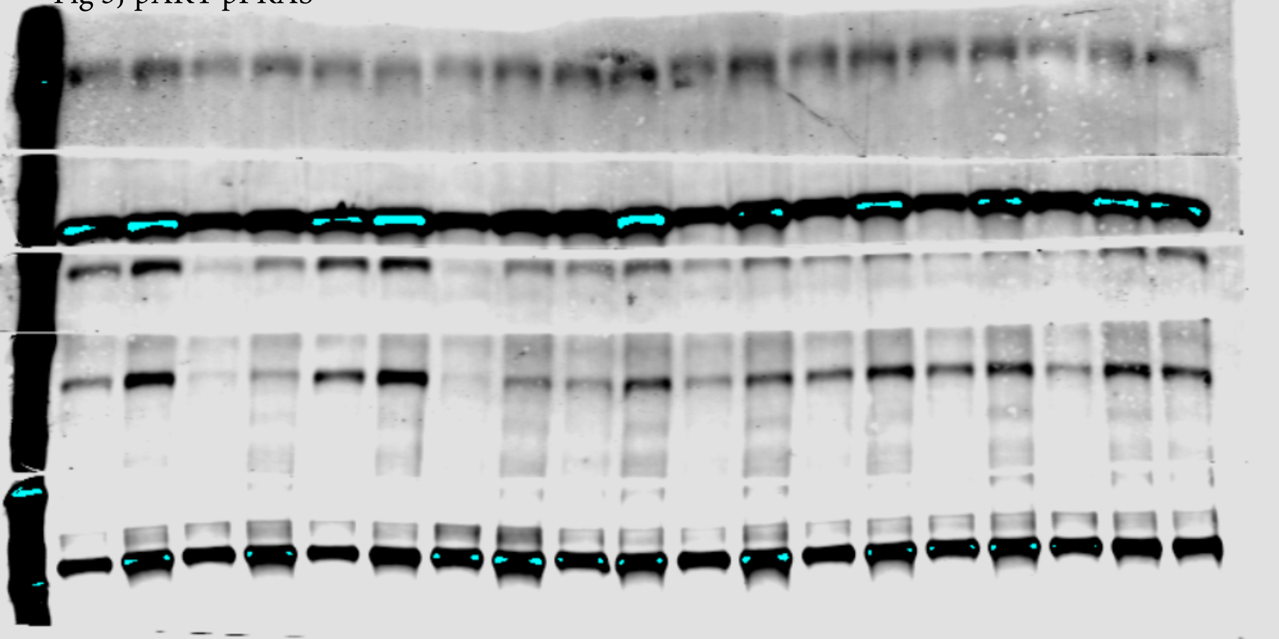

Fig 3J pEGFR

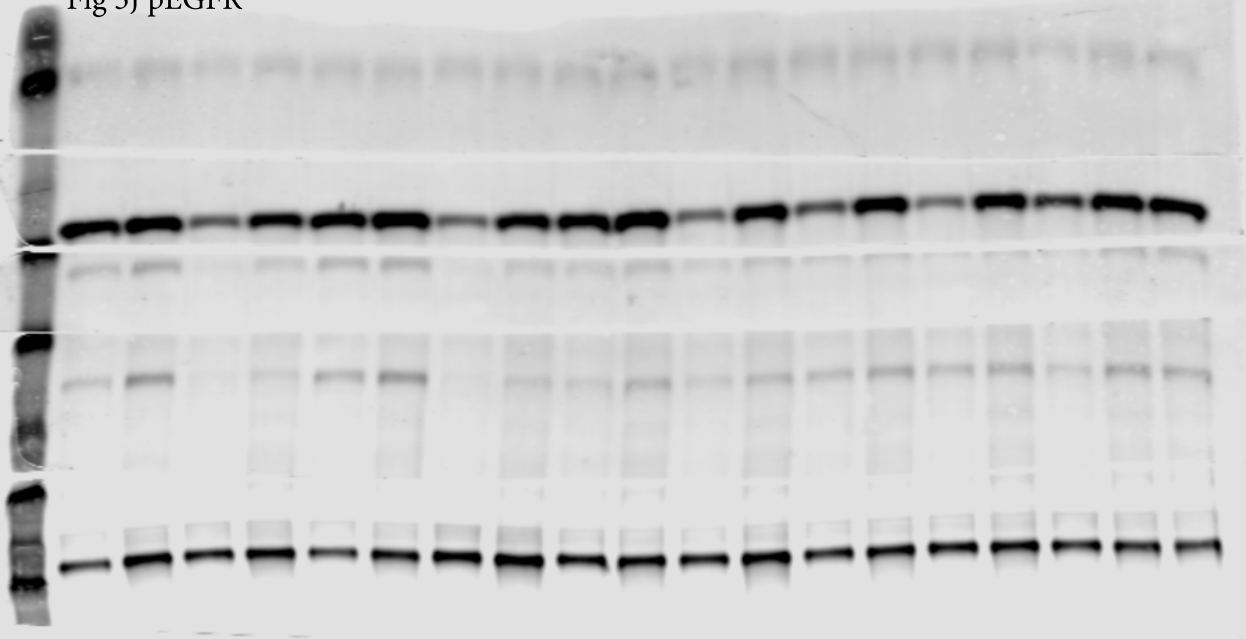

Supplement: Supplementary file 9 — Source Data for Figure 3 [file EMMM-12-e11987-s008.zip › Fig_3_part2.pdf]
