# Supplementary material for: Combined targeting of G protein‐coupled receptor and EGF receptor signaling overcomes resistance to PI3K pathway inhibitors in PTEN‐null triple negative breast cancer
Source: EMBO Mol Med. 2020 Jul 16;12(8):e11987. doi: 10.15252/emmm.202011987 (PMC7411640; doi:10.15252/emmm.202011987)

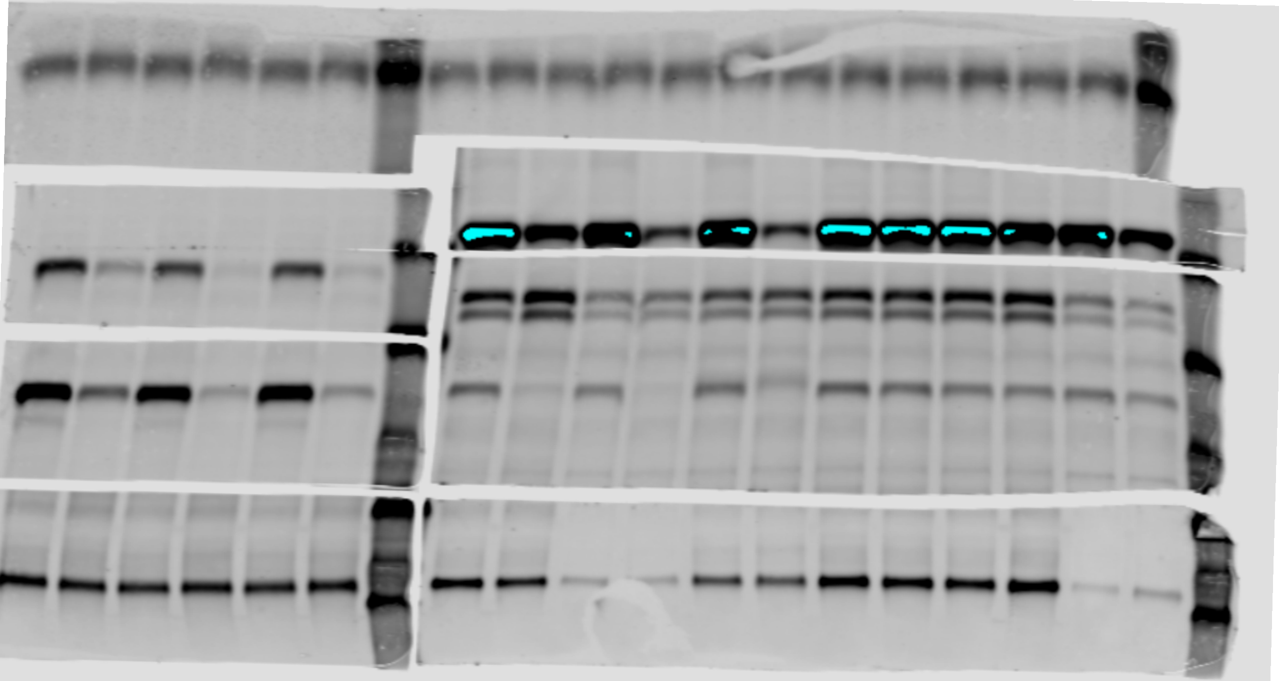

Fig 3A pEGFR_pAKT308_pERK_EGFRtot_pAKT473_pPRAS

Fig 3A pS6

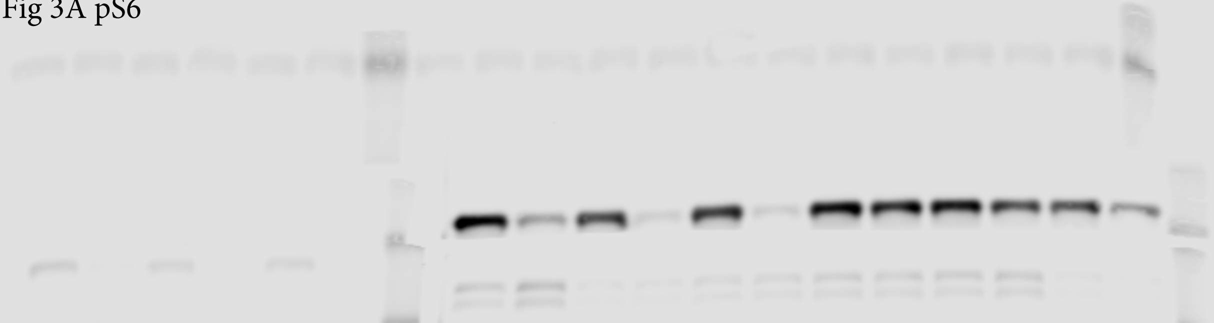

Fig 3A Vinc_AKTtot_ERKtot_S6

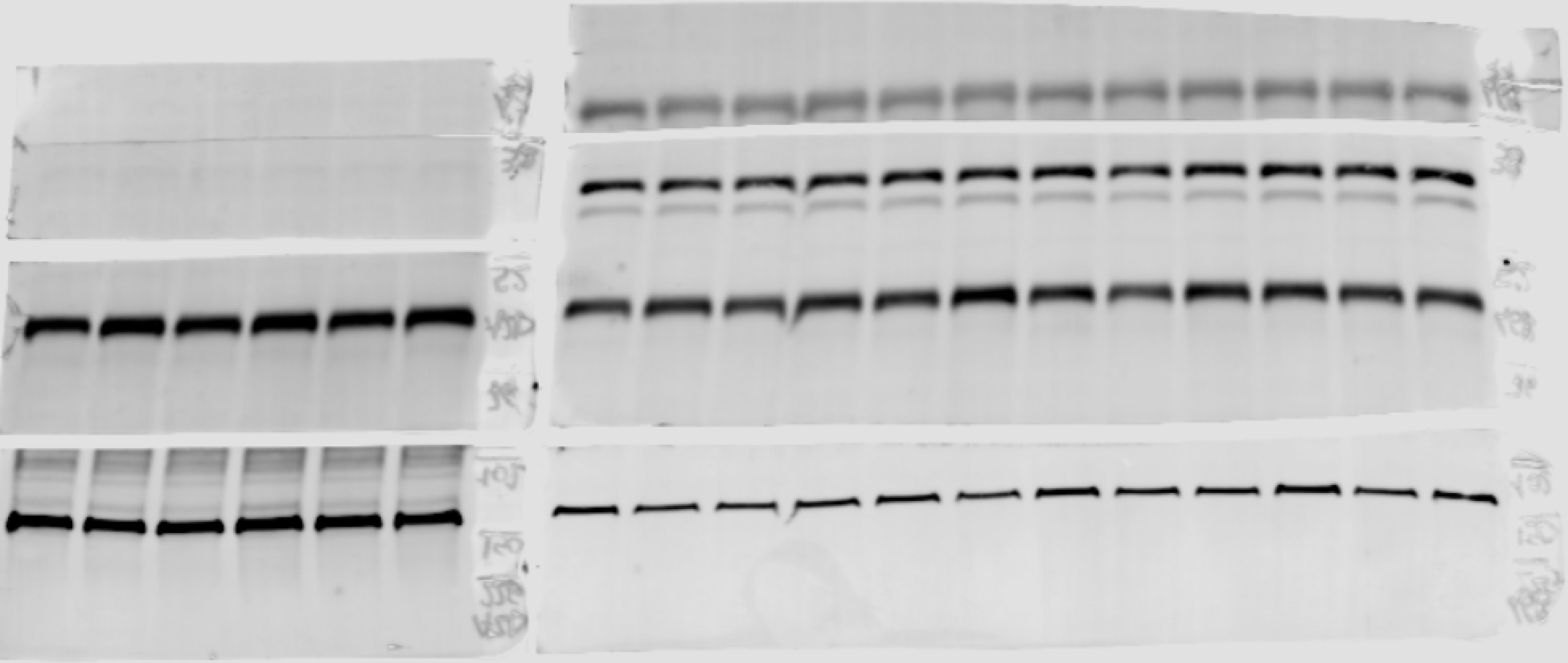

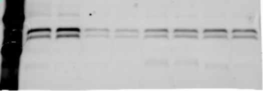

Fig 3B pERK

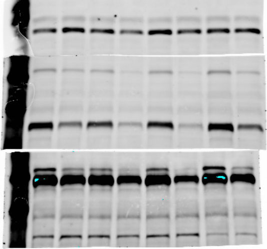

Fig 3B pS6

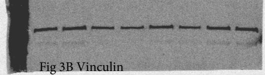
Fig 3B Vinculin

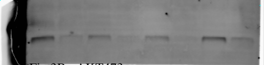

Fig.3B pAKT473

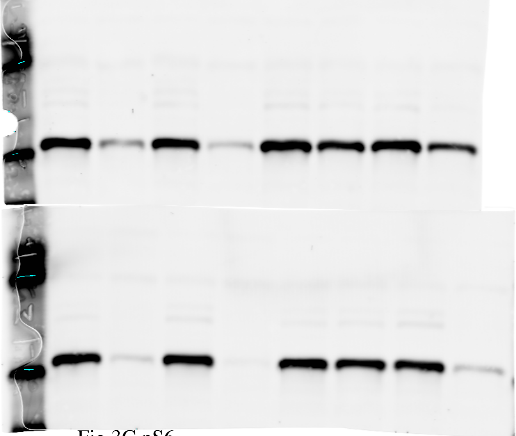

Fig 3C pS6

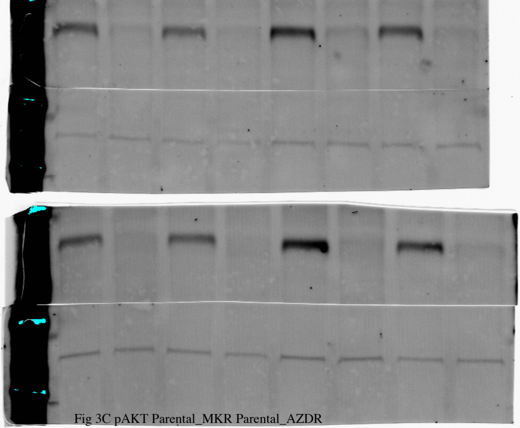

Fig 3C pAKT Parental_MKR Parental_AZDR

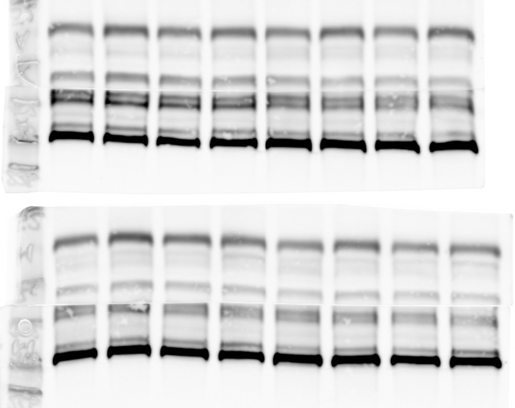

Fig 3C AKTtot Parental_MKR Parental_AZDR

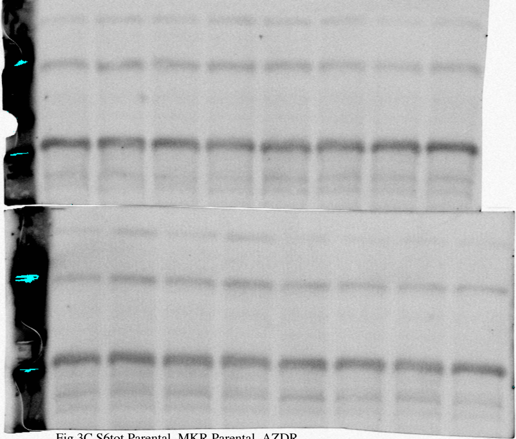

Fig 3C S6tot Parental_MKR Parental_AZDR

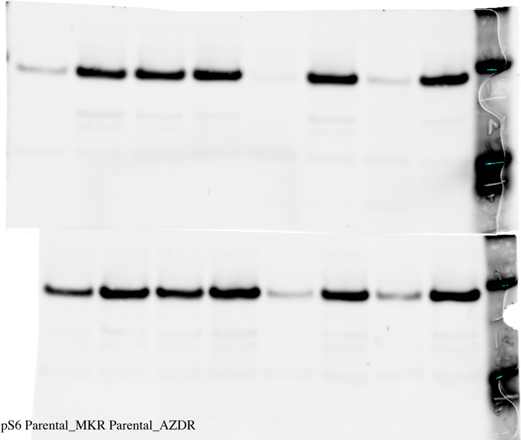

Fig 3C pS6 Parental_MKR Parental_AZDR

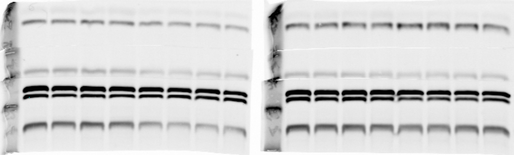

Fig 3D AKTtot ZR751

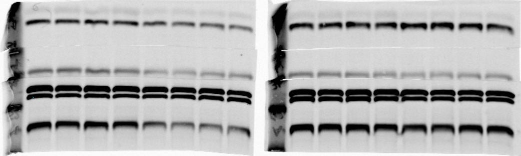

Fig 3D S6tot ZR751

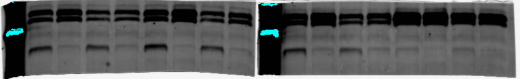

Fig 3D pAKT_ERK ZR751

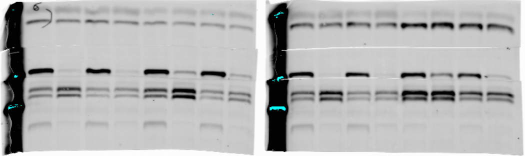

Fig 3D pAKT_ERK_S6 ZR751

Fig 3E AKTtot_left blot

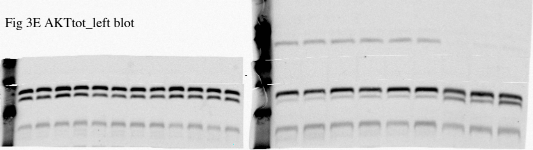

Fig 3E pAKT308_left blot

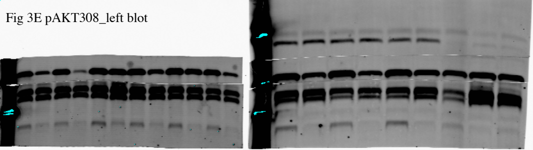

Fig 3E pS6_left blot

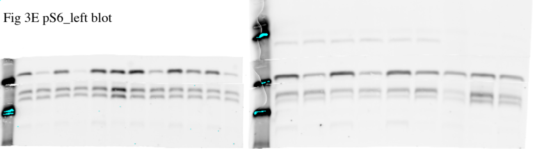

Fig 3E totS6_left blot

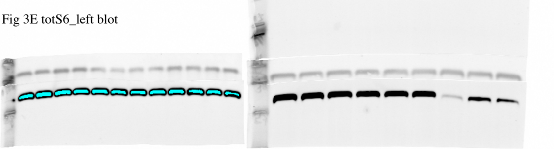

Supplement: Supplementary file 9 — Source Data for Figure 3 [file EMMM-12-e11987-s008.zip › Fig_3_part1.pdf]
