# Supplementary material for: Combined targeting of G protein‐coupled receptor and EGF receptor signaling overcomes resistance to PI3K pathway inhibitors in PTEN‐null triple negative breast cancer
Source: EMBO Mol Med. 2020 Jul 16;12(8):e11987. doi: 10.15252/emmm.202011987 (PMC7411640; doi:10.15252/emmm.202011987)

Fig 4D Vinculin

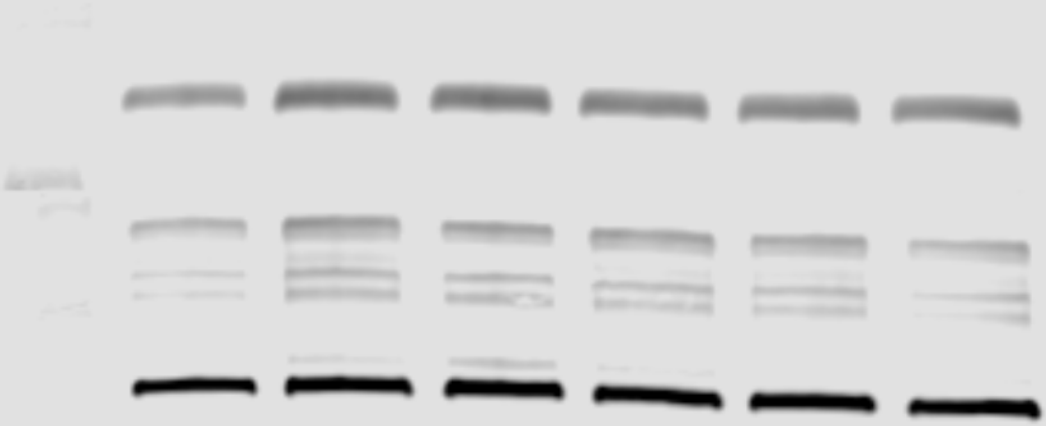

Fig 4D EGFRtot

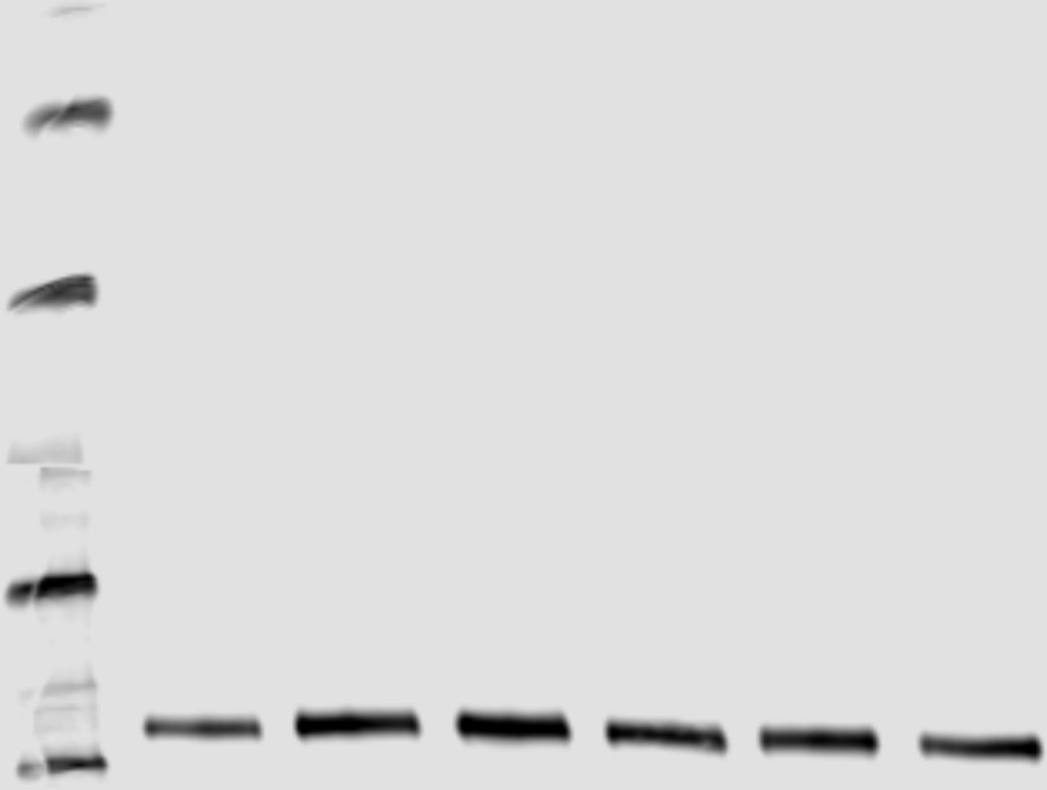

Fig 4D GNB2

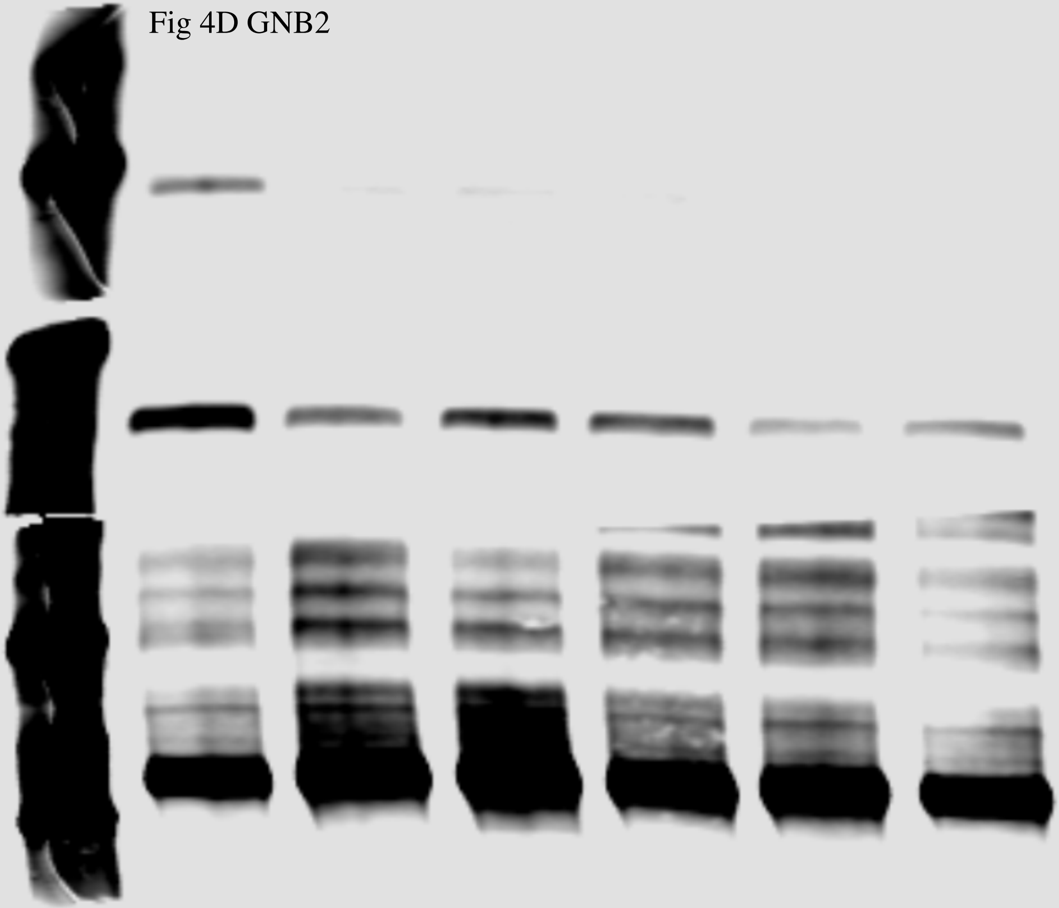

Fig 4D pAKT_right blot

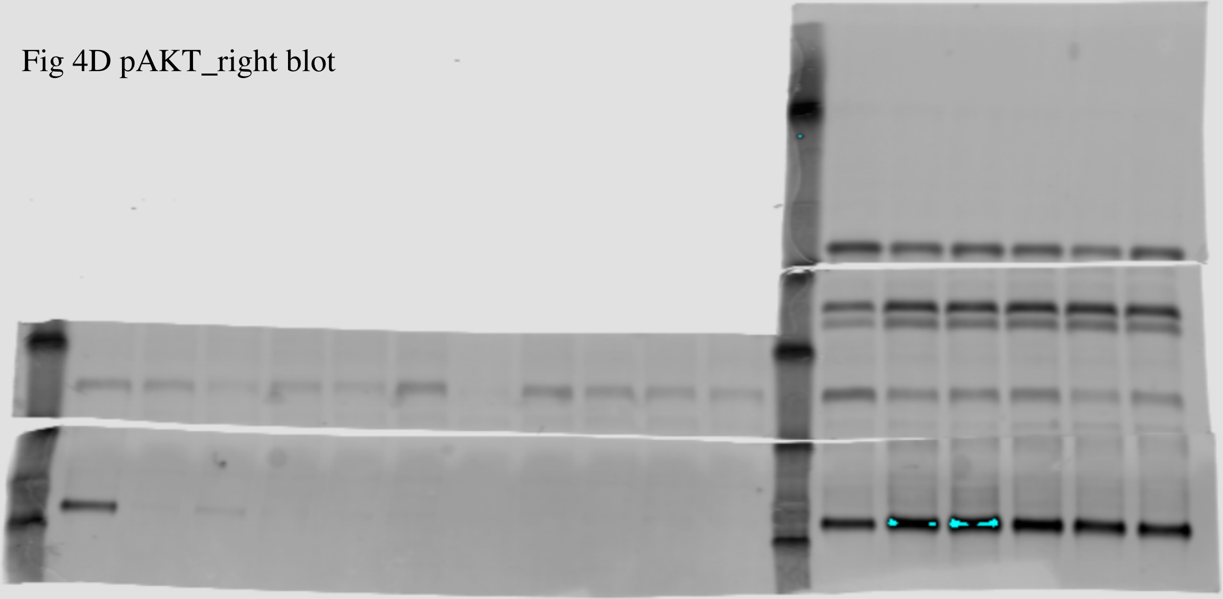

Fig 4D pEGFR pERK pS6 blot2_right blot

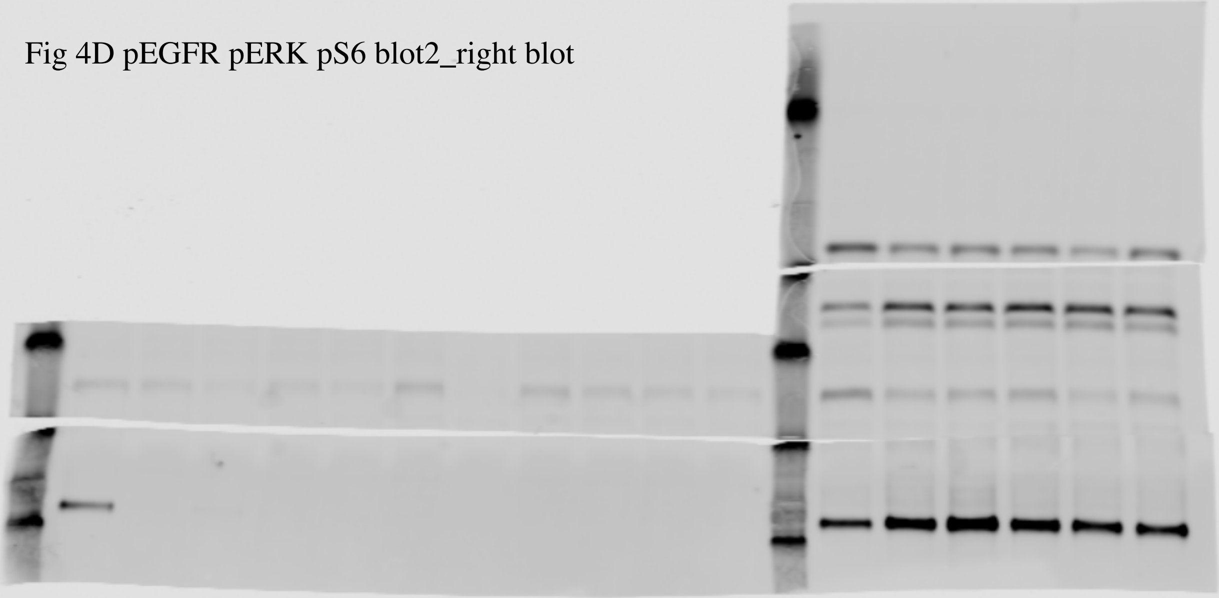

Fig 4D S6tot _right blot

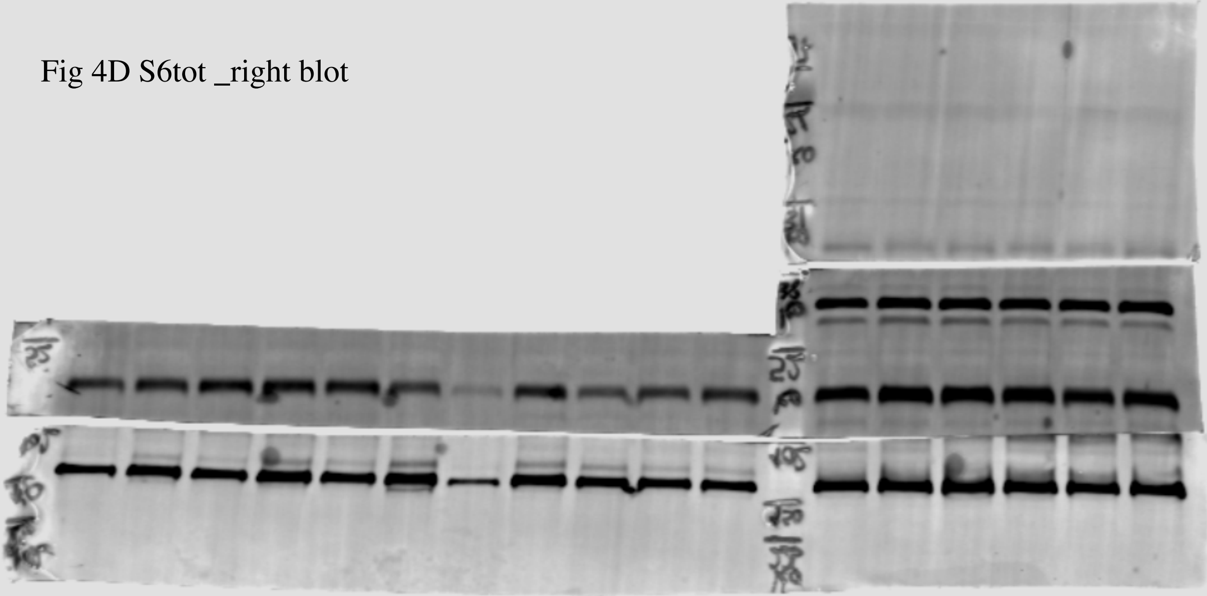

Fig 4D Vinculin AKTtot ERKtot _right blot

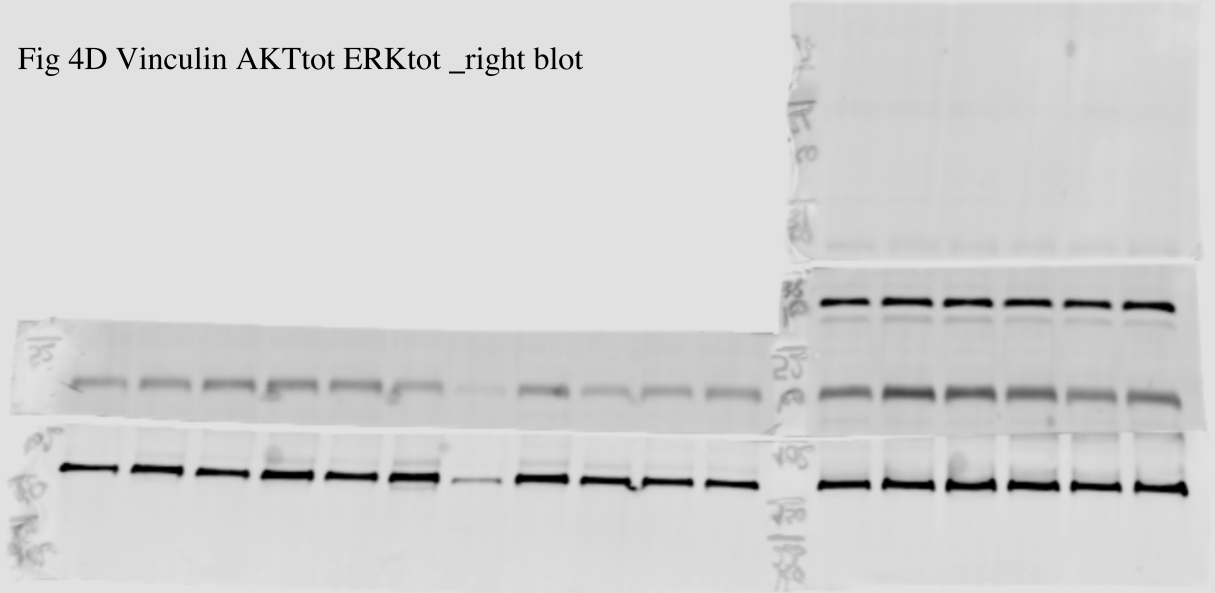

Supplement: Supplementary file 10 — Source Data for Figure 4 [file EMMM-12-e11987-s009.pdf]
