# Supplementary figures and images for: Combined targeting of G protein‐coupled receptor and EGF receptor signaling overcomes resistance to PI3K pathway inhibitors in PTEN‐null triple negative breast cancer
Source: EMBO Mol Med. 2020 Jul 16;12(8):e11987. doi: 10.15252/emmm.202011987 (PMC7411640; doi:10.15252/emmm.202011987)

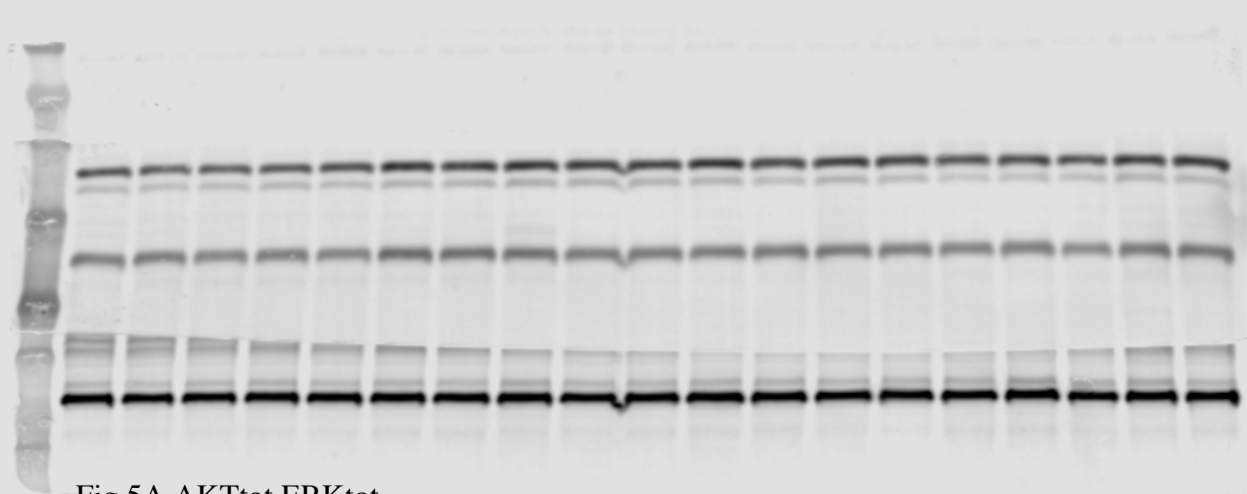

Fig 5A AKTtot ERKtot

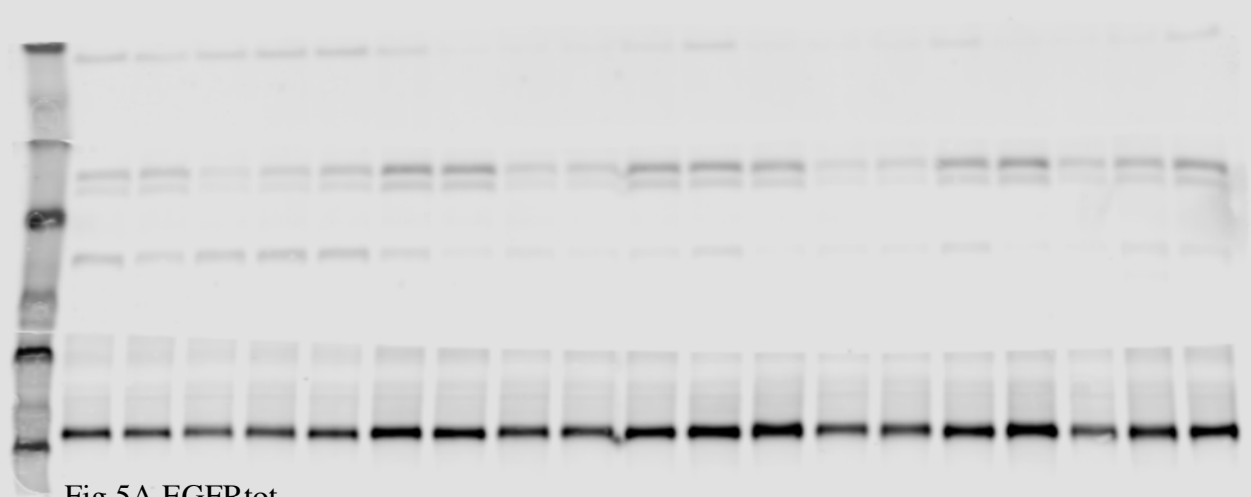

Fig 5A EGFRtot

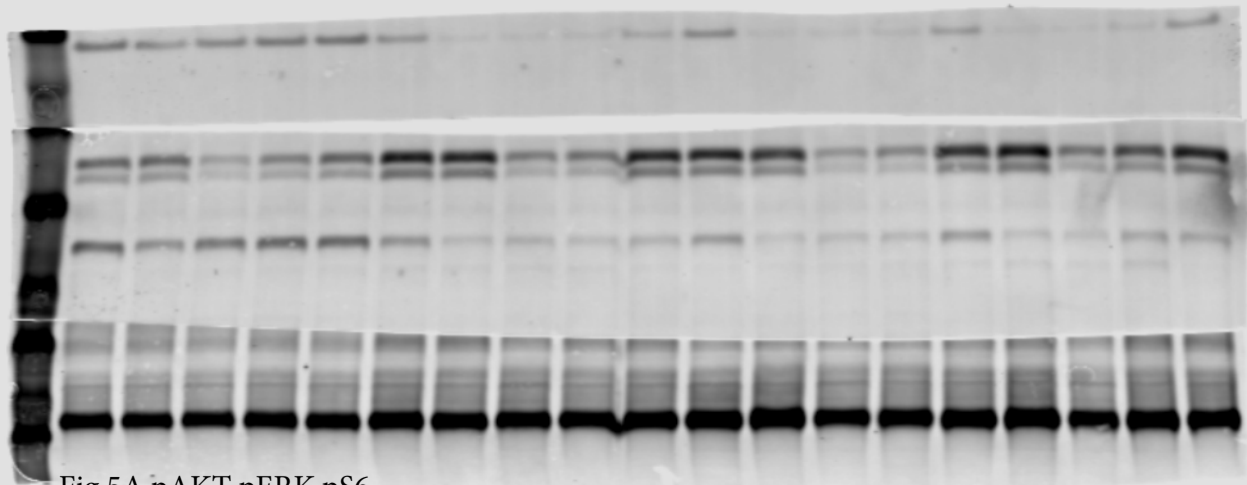

Fig 5A pAKT pERK pS6

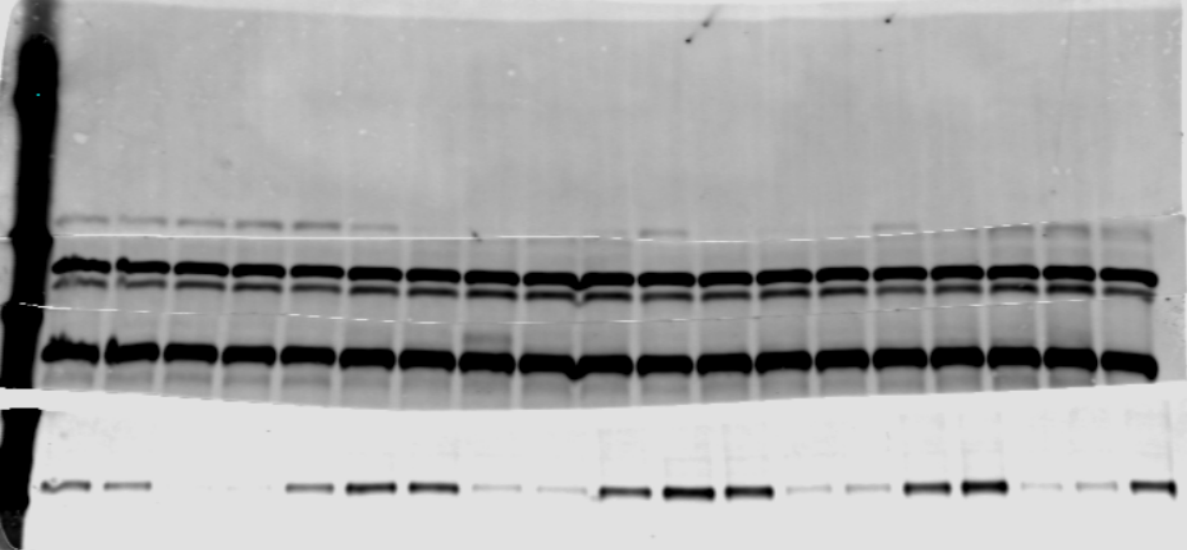

Fig 5A pEGFR

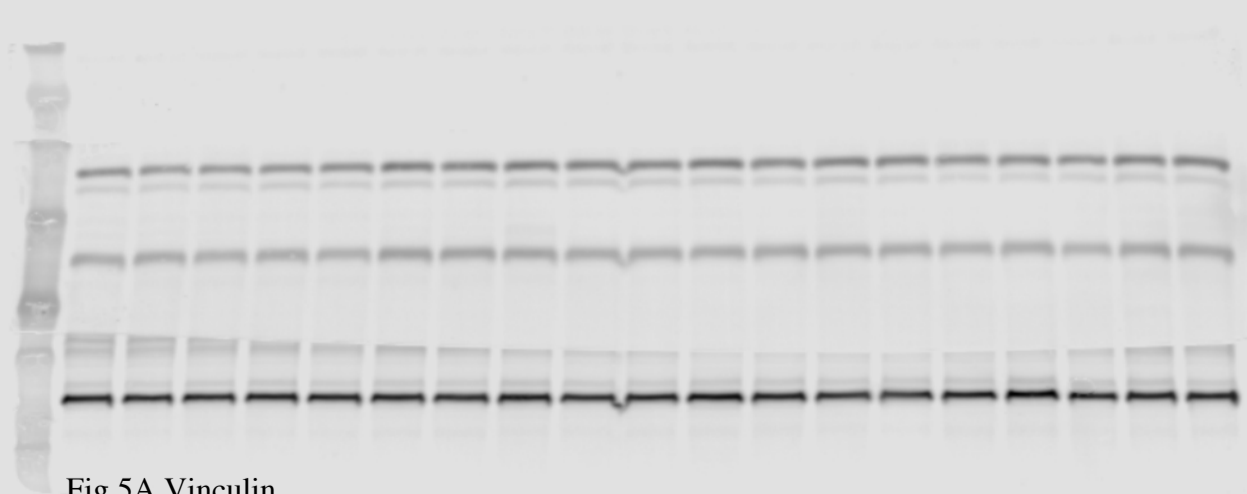

Fig 5A Vinculin

Supplement: Supplementary file 11 — Source Data for Figure 5 [file EMMM-12-e11987-s010.zip › Fig_5A.pdf]
