# Supplementary material for: Combined targeting of G protein‐coupled receptor and EGF receptor signaling overcomes resistance to PI3K pathway inhibitors in PTEN‐null triple negative breast cancer
Source: EMBO Mol Med. 2020 Jul 16;12(8):e11987. doi: 10.15252/emmm.202011987 (PMC7411640; doi:10.15252/emmm.202011987)

Fig 6B vinculin

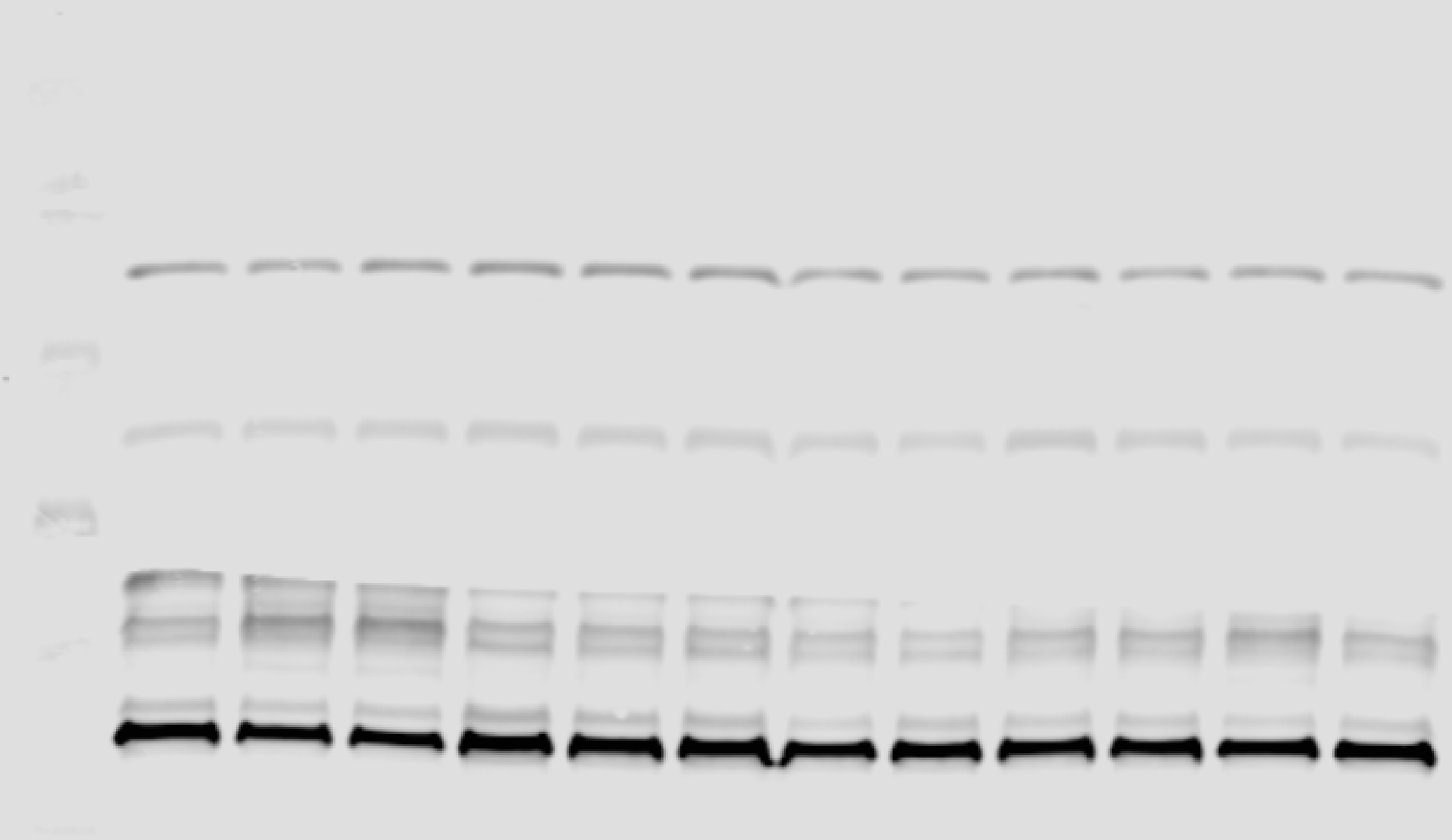

Fig 6B pEGFR_pAKT_pS6 long exposure

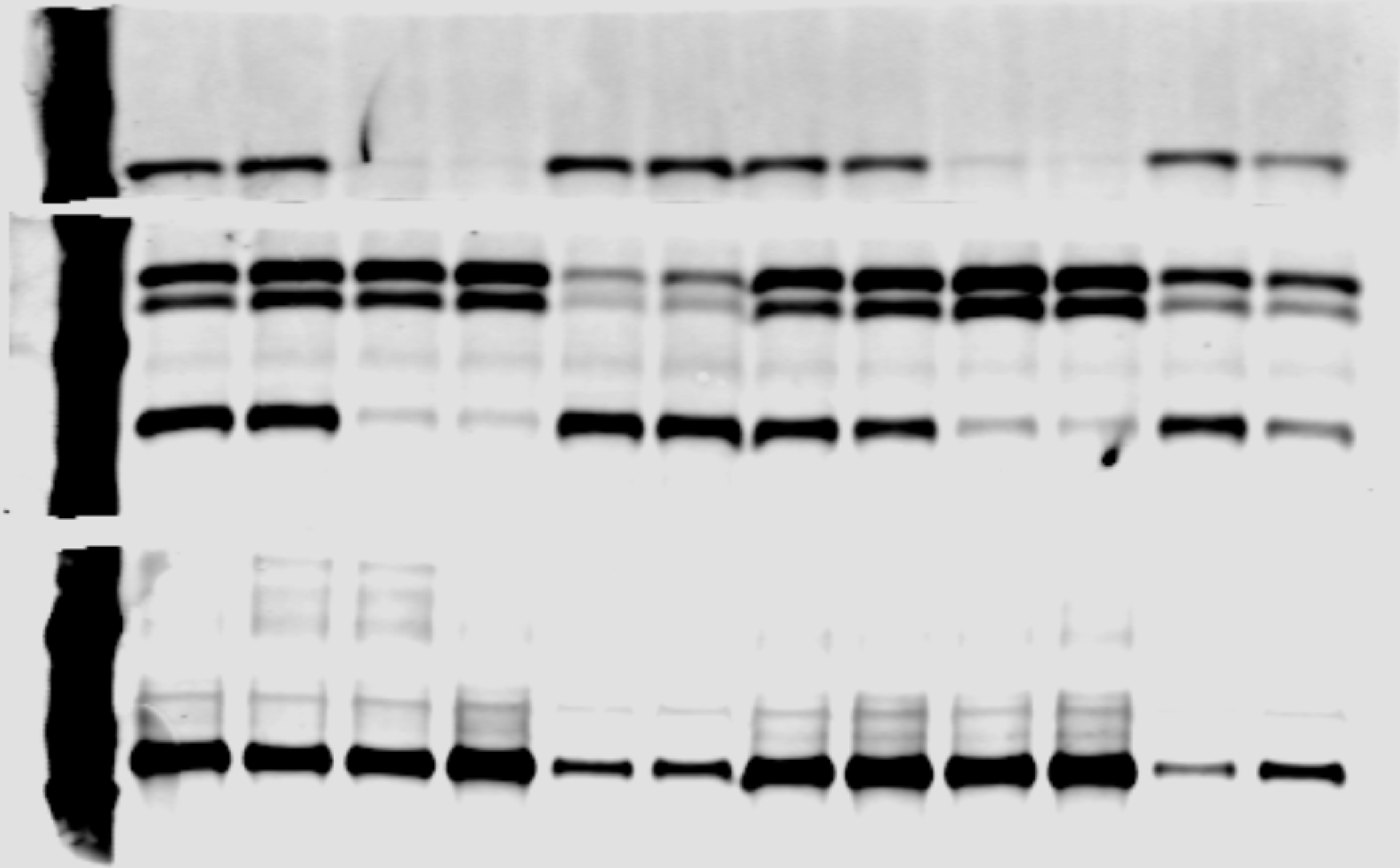

Fig 6B pEGFR_pAKT_pS6 short exposure

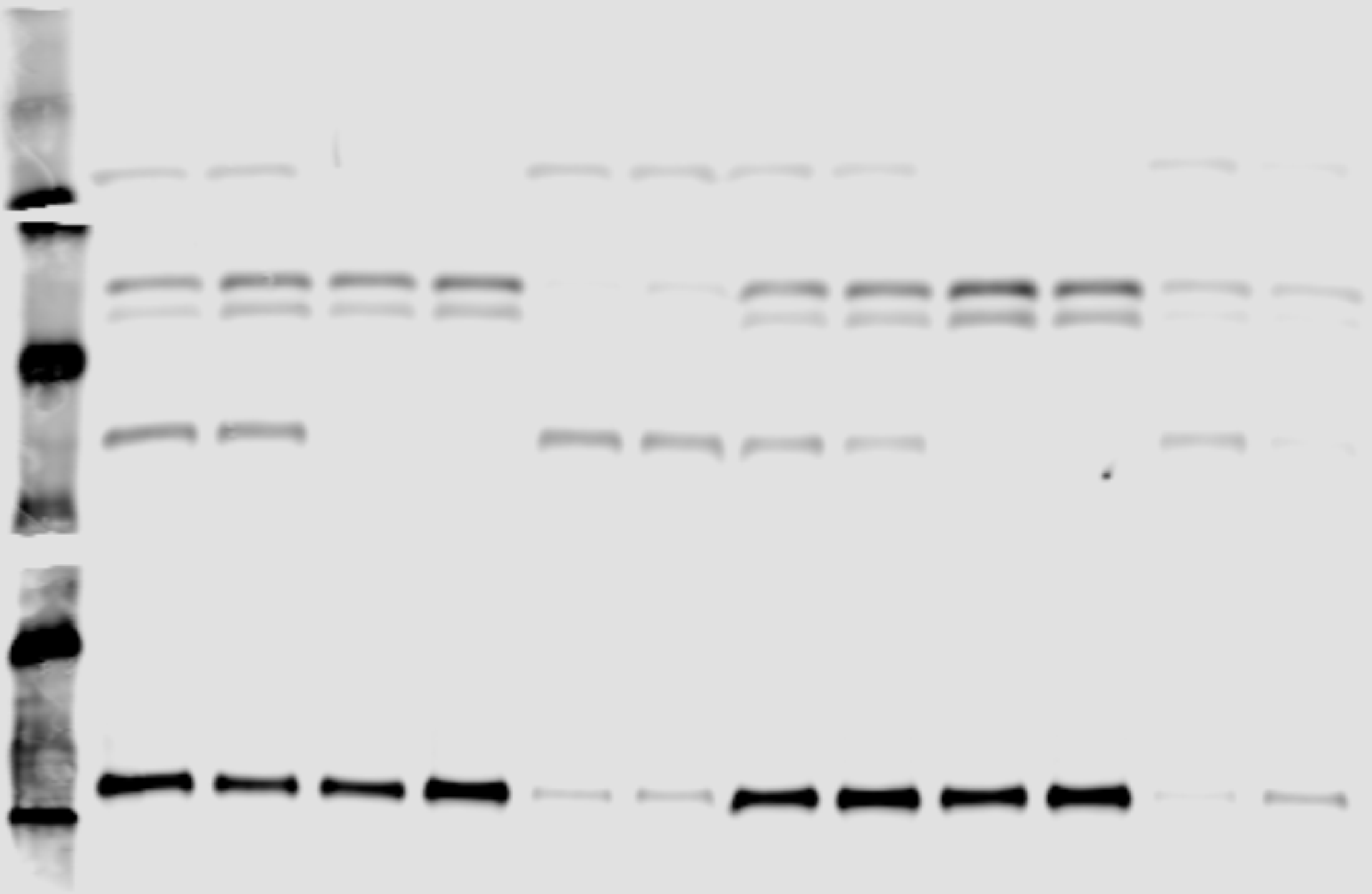

Fig 6C pEGFR_pAKT_pERK

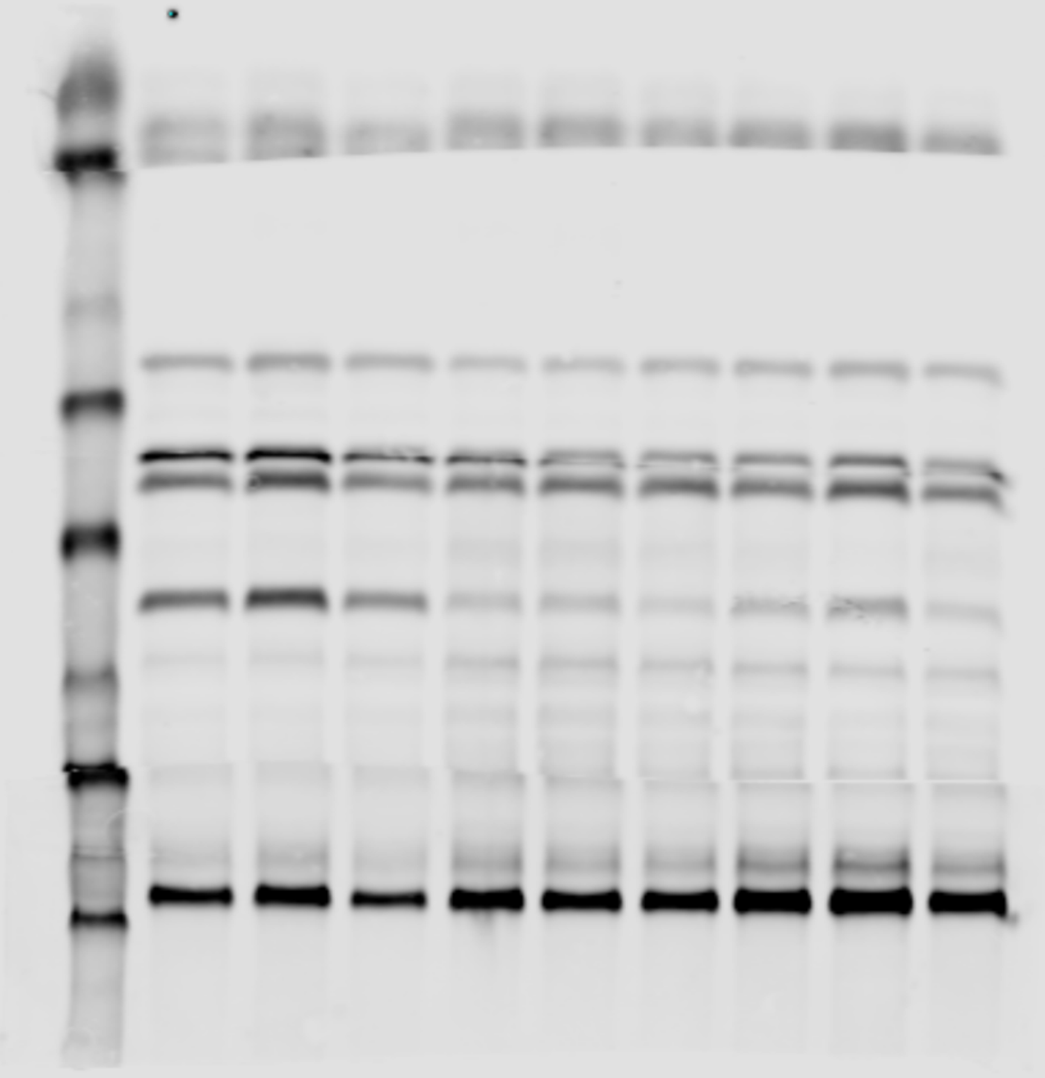

Fig 6C vinculin_AKTtot_ERKtot

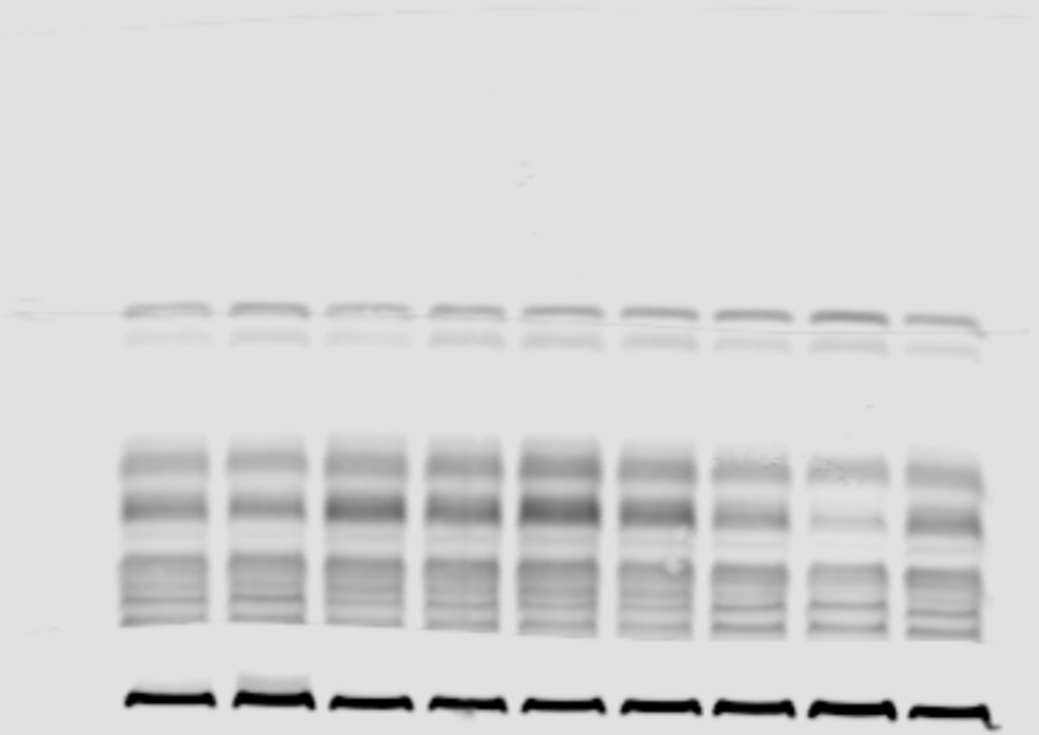

Supplement: Supplementary file 12 — Source Data for Figure 6 [file EMMM-12-e11987-s011.zip › Fig_6.pdf]
